# Sensitive and Rapid Determination of Long-Chain Per- and Polyfluoroalkyl Substances in Milk Using Fe_3_O_4_-PSA-Based Magnetic Solid-Phase Extraction Coupled with LC-MS/MS

**DOI:** 10.3390/foods15203622

**Published:** 2026-10-12

**Authors:** Tiantian Niu, Nannan Wu, Ranran Shi, Zhenzhen Liu, Siying Che, Xinquan Wang, Peipei Qi

**Affiliations:** 1 College of Chemical Engineering, Zhejiang University of Technology, Hangzhou 310014, China; 221124010424@zjut.edu.cn; 2 State Key Laboratory for Quality and Safety of Agro-Products & Institute of Agro-Product Safety and Nutrition, Zhejiang Academy of Agricultural Sciences, Hangzhou 310021, China; wunannan4089@163.com (N.W.); srr15063710928@163.com (R.S.); 18768479497@163.com (Z.L.); wangxinquan212@163.com (X.W.)

**Keywords:** per- and polyfluoroalkyl substances (PFASs), milk, Fe_3_O_4_-PSA nanoparticles, LC-MS/MS

## Abstract

A sensitive and time-saving method was developed for the determination of 11 long-chain per- and polyfluoroalkyl substances (PFASs) in milk using magnetic solid phase extraction (MSPE) coupled with liquid chromatography-tandem mass spectrometry (LC-MS/MS). A primary–secondary amine-modified Fe_3_O_4_ (Fe_3_O_4_-PSA) sorbent was used for the extraction and enrichment of PFASs from milk. The adsorption behavior of PFASs on Fe_3_O_4_-PSA was investigated, and the experimental results suggest the involvement of electrostatic and hydrophobic interactions. Effects of sample solution pH, adsorbent amount, adsorption time, eluting solvent type, and elution time on the recovery of PFASs were investigated, and the optimal conditions were determined. The results show that the method exhibited good linearity (R^2^ > 0.9972) in the range of 0.005–20 μg/kg. The limit of detection (LOD) ranged from 0.008–0.062 μg/kg, and the limit of quantitation (LOQ) ranged from 0.0260–0.206 μg/kg. Recoveries of the 11 PFASs in spiked milk samples (0.5, 5, and 20 μg/kg) ranged from 79.4% to 118% with RSDs below 17%. Therefore, the developed method demonstrated potential for the determination of PFASs in milk samples.

## 1. Introduction

Per- and polyfluorinated alkyl substances (PFASs) belong to a broad group of chemicals comprising the perfluorocarboxylic acids (PFCAs) and perfluorosulfonic acids (PFSAs), characterized by a fully fluorinated hydrophobic carbon chain bonded to diverse hydrophilic heads [1]. PFASs possess excellent properties such as hydro-oleophobicity, high surface activity, and high-temperature resistance, making them widely used in food packaging, coatings, fire retardants, and various industrial processes [2,3,4]. According to the Organization for Economic Co-operation and Development (OECD), long-chain PFASs include perfluoroalkyl carboxylic acids (PFCAs) with seven or more perfluoroalkyl carbons and perfluoroalkane sulfonic acids (PFSAs) with six or more perfluoroalkyl carbons, and their bioaccumulation and biomagnification potential increase with increasing carbon chain length. Based on the Agricultural Statistical Yearbook 2016 and the OECD/FAO report, milk and dairy products are fundamental components of the human diet, and their consumption is increasing [5]. PFASs have been detected in milk from various countries, including Germany [6], Poland [7], China [8], the United States [9] and Italy [10,11], demonstrating their widespread occurrence in dairy products. Given the high intestinal absorption efficiency of PFASs and their potential for bioaccumulation in the human body through food, they pose a threat to human health [12]. Therefore, there is an urgent need to develop a sensitive analytical method for the reliable determination of trace PFASs in milk. At present, the detection of PFASs using high-performance liquid chromatography tandem mass spectrometry (LC-MS/MS) has reached a mature stage [13]. However, the analysis of PFASs still faces many challenges. The carbon chain coverage of PFAS homologs is wide, while their physicochemical properties are very different. The milk matrix is complex, and the concentration of PFAS is at a trace level [14]. PFASs are widely present in packaging materials, water and other environmental media, which may introduce background contamination and interfere with sample preparation and detection [15,16]. Therefore, it is particularly important to develop an efficient separation and enrichment method for the reliable determination of PFASs in milk.

Various sample-preparation techniques have been developed to improve the extraction and enrichment of PFASs prior to instrumental analysis. Conventional solid-phase extraction (SPE) has been widely applied because of its relatively simple operation and good enrichment capability, while dispersive and QuEChERS-based approaches have also been introduced for the extraction of PFASs from complex food and environmental matrices [17,18]. For example, Taylor and Sapozhnikova reported a QuEChERS-based method for the determination of PFASs in foods by liquid chromatography–high-resolution mass spectrometry (LC-HRMS) and liquid chromatography–triple quadrupole mass spectrometry (LC-QqQ-MS), demonstrating the applicability of QuEChERS-based workflows to broad-spectrum PFAS analysis [19]. However, these conventional approaches may require multiple centrifugation, filtration, evaporation, or solvent-exchange steps, which can increase the sample-preparation time and may result in analyte loss or matrix effects. Recent studies have therefore focused on developing more efficient and selective sorbent-based extraction strategies. In particular, MSPE has attracted increasing attention for PFAS analysis because magnetic sorbents can be rapidly separated from complex sample matrices using an external magnetic field [20]. The development of functionalized magnetic nanomaterials has further expanded the potential of MSPE for PFAS enrichment. For instance, fluorine-functionalized magnetic amino microporous organic networks have recently been developed for the selective enrichment of PFASs, while magnetic amino-functionalized graphene oxide has also demonstrated effective adsorption of both long- and short-chain PFASs through combined electrostatic and hydrophobic interactions [21]. These advanced sorbents can introduce specific functional groups or tunable surface properties, thereby enhancing interactions with PFASs and improving extraction performance. Nevertheless, the applicability of such materials to the rapid extraction of trace long-chain PFASs from complex food matrices, particularly milk, remains insufficiently explored.

Among various functionalized magnetic adsorbents, amino-modified materials are particularly promising for PFAS adsorption due to their ability to interact with the anionic and hydrophobic moieties of PFAS molecules. As an adsorbent, magnetic materials can facilitate rapid phase separation under an applied magnetic field. By grafting appropriate functional groups onto the surface of magnetic Fe_3_O_4_, trace analytes in the sample matrix can be captured efficiently, enabling a simple and rapid separation–enrichment process. Based on the structure of PFASs, amino-modified magnetic adsorbents are effective for their adsorption [22]. The negatively charged hydrophilic groups (SO_3_^−^ and COO^−^) of PFASs enable electrostatic interactions with positively charged adsorbents, such as protonated amine groups, thereby facilitating effective adsorption. Hydrophobic interactions may also contribute to the adsorption of the fluorinated carbon chains onto hydrophobic regions of the adsorbent [22,23]. Additionally, hydrogen bonding may also contribute to the adsorption through interactions between the oxygen-containing head groups of PFASs and the nitrogen-containing functional groups of the amine adsorbent.

In our prior research, primary–secondary amine-modified Fe_3_O_4_ (Fe_3_O_4_-PSA) was prepared, displaying regular morphology and good adsorption performance toward organic acid compounds, and was subsequently used as a purification adsorbent for pesticide residue analysis [24]. Although amino-functionalized magnetic sorbents have been reported for PFAS extraction, the applicability of this previously developed Fe_3_O_4_-PSA material to the extraction of trace long-chain PFASs from complex milk matrices has not been systematically evaluated. Preliminary comparisons of short- and long-chain PFASs showed that Fe_3_O_4_-PSA exhibited relatively limited extraction performance for short-chain PFASs under the present MSPE conditions. In addition, several short-chain PFASs showed relatively low instrumental sensitivity. Therefore, the present study focused on long-chain PFASs. More satisfactory extraction performance and analytical sensitivity were achieved; an MSPE method based on Fe_3_O_4_-PSA coupled with LC-MS/MS was developed for the simultaneous determination of 11 long-chain PFASs, including PFCAs and PFSAs. Key experimental parameters, including sample solution pH, adsorbent amount, adsorption time, elution solvent type and elution time, were systematically optimized to improve the extraction performance of the proposed method. The analytical performance of the method was subsequently evaluated in terms of linearity, sensitivity, accuracy, and precision. The developed method demonstrated the feasibility of Fe_3_O_4_-PSA as an MSPE sorbent for the extraction of long-chain PFASs from milk.

## 2. Materials and Methods

### 2.1. Chemicals and Materials

High-purity (95–99.9%) standards of 11 PFASs and 2 isotopically labeled internal standards (ISs) were purchased from Dr. Ehrenstorfer, Inc. (LGC Standards, Teddington, UK), Toronto Research Chemicals, Inc. (North York, ON, Canada) and A Chemtek, Inc. (Barber Ave, Worcester, MA, USA). The 11 target PFASs were selected to include the four priority compounds specified by EFSA and EU monitoring recommendations (PFOA, PFNA, PFHxS, and PFOS), together with additional long-chain homologs covering both PFCAs (C8–C14) and PFSAs (C6–C10), to provide representative coverage for method optimization and validation. Names, abbreviations, molecular formulas, molecular weights and PK_a_ values for 11 PFAS test compounds and 2 ISs were shown in Table S1. HPLC-grade acetonitrile and methanol were purchased from Merck (Darmstadt, Germany). Ammonium acetate (HPLC-grade) was provided by Macklin Biochemical Co., Ltd. (Shanghai, China). Sodium chloride (NaCl) was purchased from Macklin Biochemical Co., Ltd. (Shanghai, China). Ultrapure water with a resistivity of 18.2 MΩ·cm at 25 °C was produced using a Milli-Q water purification system (Millipore, Billerica, MA, USA) and was used throughout the experiments. Fe_3_O_4_-PSA NPs were synthesized according to a published method in our lab. Samples of milk were obtained from a supermarket in the neighborhood (Hangzhou, China). The nutritional composition information (protein and fat content) of the 30 milks was shown in Table S2.

### 2.2. Milk Sample Pretreatment Procedure

A total of 2.0 g of milk and 20 μL of ISs standard solution (1 μg/mL) were diluted with 2.0 mL of ultrapure water in a 15 mL centrifuge tube. Then, acetonitrile containing formic acid (0.1% *v*/*v*, 2 mL) was added to the milk sample, and the centrifuge tube was vortexed for 30 s. A total of 1.0 g NaCl was added to the centrifuge tube, and the sample was vortexed for 1 min. The PP centrifuge tube was centrifuged at 6000 rpm for 1 min. The supernatant was frozen at −20 °C for 1 h and filtered. The sample solution (1 mL) was added to 19 mL of water, yielding a 20-fold dilution.

### 2.3. MSPE Procedure

The material synthesis process is based on our previous research [24], and the detailed process is shown in Text S1. A total of 20.0 mg of Fe_3_O_4_-PSA was weighed and added to the diluted solution above. The sample was vortexed for 10 min, and then the sorbent was magnetically separated and eluted with 2.0 mL of acetone under sonication for 30 s. After the magnetic separation, the eluate was filtered with a nylon membrane (0.22 μm) and evaporated under an N_2_ stream. The residues of PFASs were redissolved with 200 μL of methanol for LC-MS/MS detection.

### 2.4. LC-MS/MS Analytical Conditions

The concentrations of PFASs in the samples were determined using LC-MS/MS. The LC-MS/MS system utilized in the experiments consisted of a Shimadzu Nexera X2 series LC pump (Shimadzu Corporation, Kyoto, Japan) coupled to a Shimadzu 8050 triple-quadrupole mass spectrometer (Shimadzu Corporation, Kyoto, Japan). A Phenomenex Luna Omega C18 (Phenomenex, Torrance, CA, USA) analytical column (100 mm × 2.1 mm, 1.6 μm) was employed for chromatographic separation at 35 °C. The mobile phase consisted of water and 10% methanol (*v*/*v*) with 10 mmol ammonium acetate (phase A) and methanol with 10 mmol ammonium acetate (phase B), using a gradient elution procedure. The flow rate was 0.25 mL/min. The initial volume ratio of phase B was 10%, increased to 100% at 10 min and kept at that level until 14 min, then returned to the initial conditions at 20 min. A 10 μL aliquot was analyzed. ESI-MS/MS detection was used in negative ionization mode. The electrospray had a voltage of 4.0 kV, and the temperature of the capillary was 300 °C. The flow rates of dry gas (nitrogen), heated gas (air) and atomized gas (nitrogen) were 10.0 L/min, 10.0 L/min, and 3.0 L/min, respectively. The collision gas was argon. Mass spectrometry parameters for 11 PFAS test compounds and 2 ISs were shown in Table 1.

### 2.5. Quality Control and Method Validation

#### 2.5.1. Quality Control

The concentrations of the 11 PFASs were quantified using the internal standard calibration method [25]. Among the 11 target PFASs, ^13^C_2_-PFDA was the internal standard to calibrate PFOA, PFNA, PFDA, PFUnDA, PFDoDA, PFTrDA and PFTeDA. ^13^C_4_-PFOS was used as the internal standard for the remaining 4 PFASs (PFHxS, PFHpS, PFOS, PFDS). Although only two isotope-labeled internal standards were used, they were assigned according to the structural classes of the target analytes, with ^13^C_2_-PFDA used for PFCAs and ^13^C_4_-PFOS used for PFSAs. The use of a limited number of structurally related isotope-labeled compounds as surrogate internal standards for multiple PFASs has also been adopted in previously reported milk analysis methods [26]. Nevertheless, this approach may not fully compensate for analyte-specific differences in extraction recovery, chromatographic behavior, or ionization efficiency compared with the use of individual isotope-labeled analogs for each analyte. However, the acceptable recoveries, precision, and limited matrix effects obtained in the present study indicate that the selected internal-standard strategy provided satisfactory quantitative performance under the optimized conditions. Individual PFAS stock solutions were prepared in methanol, and mixed working standard solutions were prepared by appropriate dilution with methanol. The standard solutions were stored at 4 °C for subsequent use. Due to the pervasive presence of PFASs in the environment, experimental apparatus and the LC system components, such as the mobile phases, pipes, and other parts of the LC flow pathway (e.g., seals), may introduce significant background levels. This phenomenon can lead to a reduction in the accuracy of trace PFAS analysis. To minimize potential contamination, fluoropolymer materials, such as polytetrafluoroethylene (PTFE) tubes, were replaced with stainless steel tubing, and Teflon centrifuge tubes were replaced with low-density polyethylene (LDPE) tubes during sample preparation and instrumental analysis. All glassware was thoroughly cleaned and heated at 200 °C before use. Moreover, matrix blank, solvent blank and matrix-matching standards were injected every 10 samples for all tests. During the real sample analysis, recovery experiments were also required for each batch to control the method accuracy and precision.

#### 2.5.2. Method Validation Procedures

The method was validated by studying the linearity, LOD, LOQ, matrix effects (ME), recovery, and precision. Solvent standard solutions and milk matrix-matched standard solutions were prepared in the concentration range of 0.005 μg/L–20 μg/L, respectively. LOD and LOQ were defined using the 3-fold and 10-fold signal-to-noise (S/N) ratios based on the solvent standard solution response, respectively. The ME was determined by comparing the slope ratio of the matrix-matched calibration curve to the solvent standard calibration curve. When the value was between 0.8 and 1.2, the matrix suppression or enhancement effect was weak; when the slope ratio ranged from 0.5 to 0.8 or from 1.2 to 1.5, it was considered to indicate a moderate matrix effect; if the value was below 0.5 or above 1.5, it indicated a strong matrix effect [27].

The concentrations, recoveries and relative standard deviations (RSDs, %) of this method were calculated by analyzing repeated samples (n = 5) at three different concentration levels (0.5, 5, and 20 μg/kg). RSDs of the analysis at three concentration levels were used to evaluate the precision of the method. The reproducibility of the method was evaluated by intra-day and inter-day precision (run-to-run over 1 and 5 days).

Measurement uncertainty was estimated using a simplified Nordtest single laboratory approach. (Equations (1)–(4)). The combined standard uncertainty (*u_c_*) was calculated from the uncertainty associated with intermediate precision, *u*(*R_w_*) and the uncertainty associated with method bias, *u*(bias), according to:

(1) $$u_{c}=\sqrt{u{\left(R_{w}\right)}^{2}+u{\left(bias\right)}^{2}}$$

The intermediate precision component, *u*(*R_w_*), was estimated from the inter-day precision obtained from repeated analyses of spiked milk samples. The bias component was estimated from the deviation of the mean recoveries from the nominal spiked values at the three validation levels (0.5, 5, and 20 μg/kg). For each concentration level, the relative bias was calculated as:

(2) $$bias_{i}={\bar{R}}_{i}-100$$

where *R_i_* is the mean recovery (%) at the ith spiking level. The uncertainty associated with bias was then estimated as the root mean square of the relative biases:

(3) $$u\left(bias\right)=\sqrt{\frac{\sum_{i=1}^{n}{\left({\bar{R}}_{i}-100\right)}^{2}}{n}}$$

The expanded uncertainty (*U*) was calculated using a coverage factor of *k* = 2:

(4) $$U=ku_{c}=2u_{c}$$

### 2.6. Data Processing

Analysis of variance (ANOVA) followed by the least significant difference (LSD) post hoc test was performed using SPSS Statistics software (version 27.0.1, IBM Corporation, Armonk, NY, USA). A *p*-value < 0.05 was considered statistically significant. Different lowercase letters in the figures indicate significant differences among treatments. Graphs were prepared using Origin 2024 (OriginLab Corporation, Northampton, MA, USA), and error bars represent the standard deviations of the measurements.

## 3. Results and Discussion

### 3.1. Adsorption Performance and Mechanism of Fe_3_O_4_-PSA NPs for PFASs

#### 3.1.1. Adsorption Kinetics

The adsorption performance of Fe_3_O_4_-PSA NPs for 11 types of long-chain PFASs, including PFOA, PFNA, PFDA, PFUnDA, PFDoDA, PFTrDA, PFTeDA, PFHxS, PFHpS, PFOS, and PFDS, was investigated. As displayed in Figure 1A, Fe_3_O_4_-PSA NPs showed excellent adsorption capacity for PFAS, except for PFHxS; the adsorption capacity of the other 10 PFASs was 32.8 mg/g–40 mg/g. Meanwhile, it was also found that the adsorption capacity of PFASs increased with the increase in hydrophobicity and chain length, which is in good agreement with literature reports [28,29]. For compounds with the same carbon chain length, PFSAs showed better sorption compared with PFCAs. This can be attributed to two factors: (i) the stronger acidity and greater degree of ionization of sulfonate groups compared with carboxylate groups may favor stronger electrostatic interactions with protonated amine groups; and (ii) the higher hydrophobicity of PFSAs may enhance hydrophobic interactions with the sorbent surface [22]. These results suggest that both PFAS molecular structure and hydrophobicity influence their adsorption behavior on Fe_3_O_4_-PSA NPs.

To further investigate the adsorption behavior of these 11 PFASs on Fe_3_O_4_-PSA NPs, pseudo-first-order models (PFO) and pseudo-second-order models (PSO) were employed to fit the adsorption kinetic data obtained under optimal conditions. The fitted curves of the pseudo-first-order (PFO) and pseudo-second-order (PSO) models for the adsorption of the 11 target PFASs on Fe_3_O_4_-PSA NPs are presented in Figure 1B–L, and the corresponding kinetic parameters are summarized in Table S3.

As shown in Table S3, the R^2^ values ranged from 0.860 to 0.997 for the PFO model and from 0.757 to 0.998 for the PSO model, indicating that the applicability of the two models varied among the target compounds. In general, the PFO model yielded slightly higher R^2^ values for most PFCAs and PFHxS, whereas the PSO model showed slightly higher R^2^ values for PFOS, PFDS, PFDoDA, and PFTrDA. However, given that both models provided relatively high R^2^ values for most PFASs, and the differences between the two models were modest for several compounds, these fitting results were used primarily to characterize the adsorption kinetic behavior of different PFASs rather than to distinguish between physical and chemical adsorption mechanisms.

#### 3.1.2. Adsorption Isotherms

The adsorption isotherm data were fitted using the nonlinear Langmuir and Freundlich models, and the detailed equations and calculation procedures are provided in Text S2. As shown in Figure 2, the Langmuir model provided a better fit to the adsorption isotherm data than the Freundlich model, with (R^2^) values of 0.9546 and 0.8519, respectively. The adsorption capacity increased rapidly at low equilibrium concentrations and gradually approached a plateau at higher concentrations. The fitted maximum adsorption capacity (q_m_) obtained from the Langmuir model was 64.33 mg/g. These results indicate that PFAS adsorption on Fe_3_O_4_-PSA exhibited a saturation-type behavior, consistent with the progressive occupation of a finite number of accessible adsorption sites on the sorbent surface.

#### 3.1.3. Thermodynamic Analysis

To further investigate the thermodynamic characteristics of PFAS adsorption on Fe_3_O_4_-PSA, equilibrium adsorption experiments were conducted at three temperatures (288.15, 298.15, and 308.15 K). The equilibrium concentration (*C*e) and adsorption capacity (*q*e) obtained at each temperature were used to evaluate the temperature dependence of the adsorption process. The corresponding equilibrium constant (K) and thermodynamic parameters, including the Gibbs free energy change (Δ*G*), enthalpy change (Δ*H*), and entropy change (Δ*S*), were subsequently calculated. The detailed equations and calculation procedures are provided in Text S3.

The thermodynamic parameters calculated at different temperatures are summarized in Table S4. The negative Δ*G* values at 288.15–308.15 K indicate that PFAS adsorption onto Fe_3_O_4_-PSA was thermodynamically favorable over the investigated temperature range. The gradual increase in Δ*G* toward zero with increasing temperature suggests that adsorption became less favorable at higher temperatures. The negative Δ*H* value (−48.88 kJ/mol) confirms the exothermic nature of the adsorption process, while the negative Δ*S* value (−158.56 J/mol·K) suggests decreased randomness at the solid-solution interface during adsorption. The thermodynamic results indicate that PFAS adsorption onto Fe_3_O_4_-PSA is exothermic and thermodynamically favorable at the investigated temperatures.

#### 3.1.4. FTIR and XPS Characterization

The FTIR spectra and high-resolution XPS spectra of Fe_3_O_4_-PSA before and after PFOA adsorption are shown in Figure 3. As shown in Figure 3A, the main characteristic FTIR absorption bands remained essentially unchanged after adsorption, and no obvious new absorption bands or substantial peak shifts were observed. In particular, the broad O–H/N–H stretching band around 3400 cm^−1^ showed no clear red shift or pronounced broadening. These observations indicate that the overall chemical structure of Fe_3_O_4_-PSA remained stable during the adsorption process, and the FTIR spectra alone do not provide conclusive evidence for the formation of specific hydrogen-bonding interactions.

The C 1s spectra (Figure 3B) were deconvoluted into components assigned to C-C/C-H, C-O, and N-C=O. The corresponding binding energies changed only slightly after PFOA adsorption, from 284.80, 286.27, and 287.88 eV to 284.80, 286.29, and 287.78 eV, respectively, indicating that the carbon-containing framework of Fe_3_O_4_-PSA was largely preserved. In contrast, more evident changes were observed in the N 1s spectra (Figure 3C). After PFOA adsorption, the -NH component shifted from 399.14 to 399.29 eV, while the C-N component exhibited a more pronounced positive shift from 401.05 to 401.63 eV. In addition, a new N-related component appeared at approximately 400.81 eV. These variations in the N 1s binding energies indicate a redistribution of the local electron density around the nitrogen atoms and provide evidence for the involvement of nitrogen-containing functional groups in hydrogen-bonding interactions with PFOA. Furthermore, a distinct F 1s signal at 688.48 eV was observed after adsorption (Figure 3D), which was assigned to C-F species and provides direct evidence for the presence of PFOA on the Fe_3_O_4_-PSA surface. Combined with the pH-dependent adsorption behavior and zeta-potential results, these XPS observations support the involvement of electrostatic interactions between protonated amine groups and anionic PFOA. Meanwhile, the changes observed in the N 1s spectra may support the involvement of hydrogen-bonding interactions between PFOA and the nitrogen-containing functional groups of Fe_3_O_4_-PSA, while hydrophobic interactions involving the perfluorinated carbon chain may also contribute to the adsorption process.

### 3.2. MSPE Method Development

After preliminary experiments, the milk sample pretreatment procedure was developed. Both PFCAs and PFSAs are acidic compounds that remain non-dissociated in an acidic environment, facilitating their transfer to the organic phase [30]. Therefore, acetonitrile containing formic acid (0.1%, *v*/*v*) was selected as the extraction solvent. The addition of NaCl could reduce the solubility of PFASs in water and promote their transfer to the organic phase [31]. Residual fat and protein were removed from the substrate by rapid freezing at −20 °C. After the preliminary experiments and screening, the main conditions that needed to be optimized in the MSPE procedure included the pH of the extraction solution, the amount of adsorbent material, adsorption time, the type of elution solvent and elution time.

#### 3.2.1. Effect of pH

The zeta potential measurements of the Fe_3_O_4_-PSA nanocomposite (Figure 4A) reveal a distinct pH-dependent surface charge profile. The material exhibits a positive ζ-potential at pH values below its isoelectric point (IEP) of 8.0 and a negative zeta potential above this threshold. This behavior is primarily attributed to the protonation and deprotonation of the abundant amine (–NH_2_) groups grafted onto the nanoparticle surface [24]. At low pH, the high concentration of H^+^ ions drives the protonation of –NH_2_ to –NH_3_^+^, imparting a positive charge to the adsorbent. As the pH approaches the IEP, the net surface charge approaches zero, indicating a balance between protonated and deprotonated species. Beyond pH 8.0, the progressive deprotonation of amine groups, coupled with the possible contribution of surface hydroxyl groups (–OH), results in a negatively charged surface. This transition influences the electrostatic interaction potential between the adsorbent and the anionic PFASs in aqueous solution.

Adjusting the sample solution to an appropriate pH is crucial for enhancing the extraction efficiency of the target analytes. Therefore, the extraction efficiency of Fe_3_O_4_-PSA for PFASs over a pH range of 3–10 was investigated. The recovery profile of PFASs as a function of solution pH (Figure 4B) generally corresponded to the pH-dependent variation in the surface charge of Fe_3_O_4_-PSA. At pH < 8.0 (e.g., pH 3.0–6.0), the adsorbent surface is positively charged due to the aforementioned protonation of –NH_2_ groups [24]. According to Table S1, the dissociation constants of the 11 target PFASs were all less than 3.8. Under these conditions, the anionic PFASs can interact electrostatically with the positively charged Fe_3_O_4_-PSA surface, which may contribute to their adsorption. Notably, the recoveries varied among the individual PFASs over the acidic pH range, and some compounds exhibited relatively high recoveries at pH 3.0–4.0. At the IEP (pH = 8.0), the net surface charge is close to neutral, and electrostatic attraction is substantially weakened. However, measurable recoveries were still observed, indicating that electrostatic interactions alone could not fully account for the retention behavior of PFASs on Fe_3_O_4_-PSA. When pH > 8, both the adsorbent surface and the target analytes were negatively charged, resulting in electrostatic repulsion, which is consistent with the generally decreased recoveries observed at pH 9.0–10.0. Although some individual PFASs exhibited higher recoveries at pH 4.0, pH 6.0 provided a more balanced overall recovery for the 11 target PFASs and was therefore selected for subsequent experiments.

#### 3.2.2. Effect of Adsorbent Dosage

The amount of Fe_3_O_4_-PSA adsorbent had a significant impact on the extraction efficiency of the established MSPE method, so the amount of Fe_3_O_4_-PSA adsorbent was optimized in the experiment. The effects of different amounts (5, 10, 20, 30, 40 and 50 mg) of Fe_3_O_4_-PSA adsorbent on the recoveries of 11 long-chain PFASs were investigated. As shown in Figure 5, with the increase in the amount of adsorbent from 5 mg to 20 mg, the number of long-chain PFASs within a reasonable recovery range increased from 54.5% to 63.6%. This indicated that the increase in the amount of adsorbent provided more accessible surface sites for PFAS adsorption, thereby improving the extraction of several target analytes. When the adsorbent dosage was between 30 and 50 mg, the recoveries of half of the target PFASs showed a declining trend. This decrease is attributed to nanoparticle aggregation driven by magnetic dipole–dipole interactions at high particle concentrations, which reduces effective surface area and physically entraps adsorbed long-chain sulfonates within inter-particle voids, hindering subsequent desorption [32]. Therefore, 20 mg of Fe_3_O_4_-PSA was the optimal adsorbent dosage and was used in subsequent experiments.

#### 3.2.3. Effect of Adsorption Time

The proper adsorption time was important for the effective dispersion of the adsorbent in the sample solution and the optimal adsorption performance. The effect of Fe_3_O_4_-PSA on the recoveries of the target compounds at different adsorption times (2, 5, 10, 15, 20 and 30 min) was investigated to determine the optimal adsorption time. As shown in Figure 6, the recoveries of 8 PFASs were between 70% and 120% when the adsorption time was 2–15 min. With the increase in adsorption time, the recoveries of the 11 PFASs varied from 62.2–119% to 48.4–122%. When the adsorption time was 10 min, the RSDs of all target analytes were in the range of 2.1% to 8.2%. When the adsorption time increased from 15 min to 30 min, the recoveries decreased significantly. The initial over-recovery (>100% for several analytes) may be associated with matrix-induced signal enhancement. The subsequent decrease may be related to reversible desorption or changes in the adsorption equilibrium under prolonged agitation, as reported for PFAS adsorption systems [33,34]. Short-chain homologs (e.g., PFHxS, PFNA) consistently yield low and falling recoveries due to their higher aqueous solubility and weaker hydrophobic interactions [35]. These kinetic features indicate that prolonged extraction compromises quantitative accuracy rather than improving it. The optimal extraction window is ≤10 min, which provides acceptable recoveries for most analytes while minimizing analyte loss and matrix interference. Therefore, in subsequent experiments, 10 min was selected as the optimal adsorption time.

#### 3.2.4. Effect of Elution Solvent Type

The type of elution solvent has a significant effect on the recoveries of the target analytes. In order to obtain higher recoveries, five different elution solvents (acetone, 0.1% formic acid–acetonitrile (*v*/*v*), acetonitrile, 0.1% formic acid–methanol (*v*/*v*), and methanol) were used to elute the Fe_3_O_4_-PSA adsorbent loaded with long-chain PFASs. As shown in Figure 7, when the Fe_3_O_4_-PSA was eluted with acetone, acetonitrile and 0.1% formic acid–methanol, the recoveries of the 11 PFASs were 79.0–114%, 79.7–110% and 71.4–99.6%, respectively. When 0.1% formic acid–acetonitrile or methanol was used as an elution solvent, only seven PFASs had recoveries in the 70–120% range. When acetone and acetonitrile were used to elute the adsorbent, the RSDs of the 11 PFASs ranged from 2.4–10% and 3.0–17%, respectively. Notably, acetone’s low viscosity and high diffusivity further facilitate rapid mass transfer during the desorption step. In addition, nitrogen evaporation was performed during the experiment, and the drying speed of the acetone solution was significantly faster than that of the acetonitrile solution under a nitrogen stream. Therefore, acetone was finally selected as the elution solvent.

#### 3.2.5. Effect of Elution Time

The elution time of Fe_3_O_4_-PSA adsorbent using the elution solvent was optimized. The effect of different elution times (0.5, 1, 2, 3, 4,and 5 min) on the recoveries of the 11 long-chain PFASs was investigated. The elution time profile (Figure 8) reveals that the desorption of PFASs from Fe_3_O_4_-PSA is remarkably rapid, with most analytes reaching near-maximal recoveries within 0.5–1 min. When the elution time was 0.5 min, the recoveries of the 11 target compounds ranged from 78.0% to 121%, with the RSDs ranging from 2.5% to 11%, indicating rapid desorption of PFASs from Fe_3_O_4_-PSA. This rapid desorption may be associated with the ability of acetone to weaken interactions between PFASs and the sorbent, including hydrophobic interactions and other possible intermolecular interactions reported in PFAS adsorption systems [33]. When the elution time was increased to 4 min, the recoveries of PFASs were 55.2–123%. When the elution time was 0.5–4 min, the recoveries of the PFASs showed a decreasing trend, indicating that prolonging the elution time did not further improve the overall recovery of the target PFAS. When the elution time increased to 5 min, the recoveries of the target PFAS increased significantly, and 40% of the PFASs had recoveries of over 120%. Recoveries exceeding 120% may be associated with calibration bias rather than an actual increase in elution efficiency. Therefore, the optimal time for the elution solvent to elute the target compounds from Fe_3_O_4_-PSA was 0.5 min.

Finally, after 2 g of milk was pretreated according to the procedure described in Section 2.2, 20 mg of Fe_3_O_4_-PSA was used to adsorb long-chain PFASs from the sample. After vortex adsorption for 10 min, the adsorbent was separated by magnetic separation. The adsorbent was eluted with 2 mL of acetone under ultrasonication for 30 s. The magnetic separation operation was repeated to separate the eluent from the adsorbent. The elution solution was filtered through a nylon membrane filter (0.22 μm) and purged under N_2_ flow until nearly dry. The residue was redissolved in 200 μL of methanol and analyzed by LC-MS/MS.

### 3.3. Method Validation

The method was validated by studying the linearity, LOD, LOQ, ME, recovery, and precision. The linearity of the method was evaluated with standard solutions at different concentrations (0.005, 0.01, 0.02, 0.05, 0.1, 0.2, 0.5, 1, 5, 10 and 20 μg/L). The peak area ratio of the target analyte to the corresponding internal standard was taken as the ordinate (Y) with the concentration ratio as the horizontal coordinate (X), thereby generating a fitted calibration curve for each target compound. The correlation coefficient (R^2^) was used to evaluate the linearity, and the results show that the R^2^ of the 11 PFASs ranged from 0.9972 to 0.9999 (Table 2). The LOD for the 11 PFASs ranged from 0.008 to 0.062 μg/kg and the LOQ ranged from 0.026 to 0.206 μg/kg. The LOQ obtained by the developed MSPE method in this study was lower than the 1 μg/kg recommended by the European Commission (2010/161/EU) [36] for PFOA and PFOS. As shown in Table 2, the ME of all 11 PFASs ranged from 0.9 to 1.1. Representative MRM chromatograms of PFUnDA in the matrix-matched standard, solvent standard, procedural blank, and matrix blank are shown in Figure S1. No obvious interfering peaks were observed in the procedural blank or matrix blank at the retention time of PFUnDA. The ME values indicated that matrix effects were limited under the optimized analytical conditions.

As shown in Table S5, the recoveries of 11 long-chain PFASs in milk samples were 79.4–118% and RSDs were all lower than 17% at three standard concentrations (0.5, 5 and 20 μg/kg). These results show that when the concentration of PFASs was higher than the LOQ, the developed MSPE method could accurately analyze 11 target PFASs. At the same time, intra-day RSDs were used to evaluate the reproducibility of the method. Inter-day RSDs ranged from 1.6–19%, and the recoveries were 76.3% to 120%. The experimental results show that the MSPE pretreatment method established in this study had good reproducibility.

The measurement uncertainty of the 11 PFASs was further evaluated based on the inter-day precision and method bias at three spiking levels (0.5, 5, and 20 μg/kg), following the single-laboratory approach previously applied to PFAS determination in milk [26]. The combined standard uncertainty (u_c_) ranged from 12.4% to 18.3%, and the corresponding expanded uncertainty (U,k = 2) ranged from 24.9% to 36.6% for the 11 PFASs. These results provide an additional quantitative assessment of the reliability of the developed method. Moreover, the robustness of the method is indirectly supported by the consistently low RSD values obtained from inter-day precision experiments (RSD < 20%) and the stable recoveries across different milk batches, indicating acceptable tolerance to minor operational variations.

### 3.4. Comparison with Reported Methods for PFAS Analysis

To further evaluate the analytical performance, the proposed Fe_3_O_4_-PSA-based MSPE method was compared with recently reported SPE, QuEChERS, and MSPE methods for PFAS analysis. Table 3 lists the sample amount, sorbent type and consumption, extraction time, organic solvent consumption, LOD, LOQ, and recovery, as summarized in Table 3. Compared with the conventional SPE method [37]. The proposed MSPE procedure requires fewer sample-preparation steps and lower organic solvent consumption. In comparison with recently reported QuEChERS- and MSPE-based methods, the present method also uses a relatively low amount of sorbent and a short extraction time. However, some reported methods provide comparable or lower detection limits. Therefore, the main advantages of the proposed method lie in its relatively simple operation, low sorbent consumption, and rapid magnetic separation.

The developed MSPE method exhibited obvious advantages. In terms of the amount of adsorbent, the MSPE method could achieve efficient enrichment of the 11 target PFASs with only 20 mg, while the SPE method required 500 mg of adsorbent. The volume of elution solvent required for a single sample in the SPE method was 10 mL, and the operation process was cumbersome; the recoveries of 11 target PFAS were 91–105%. The developed MSPE method required only 2 mL of elution solvent per sample, and the recoveries of 11 PFASs ranged from 79.4–118%. In contrast, the MSPE method used smaller amounts of adsorbent and organic solvent, so this method was more economical and environmentally friendly than the SPE method.

### 3.5. Real Samples Analysis

To verify the applicability of the established method, it was applied to the determination of long-chain PFASs in 30 milk samples of different types (whole milk, low-fat milk, skimmed milk and fresh milk) and from various brands. The product information for the 30 milk samples is presented in Table S2. Each milk sample was analyzed in triplicate. Results of the real sample analysis were shown in Figure 9. Among the 30 milk samples, five long-chain PFASs were detected in 14 samples (whole pure milk and fresh milk), including PFDoDA (0.032 μg/kg), PFUnDA (0.029 μg/kg), PFDA (0.033 μg/kg), PFOA (0.037–0.262 μg/kg) and PFHpS (0.031–0.069 μg/kg). PFOA showed both the highest concentration and the highest detection frequency among the target analytes, which is consistent with recent observations in Chinese raw milk [42,43]. Previous studies have reported markedly different PFAS contamination levels in milk across countries. In Poland, Sznajder-Katarzyńska et al. reported that 43.4% of the investigated milk and dairy-product samples contained PFASs above the LOQ, with PFOA being the most frequently detected compound [7]. In South Africa, the total concentration of 15 PFASs (Σ15PFAS) in retail dairy milk ranged from 0.08 to 15.51 ng·mL^−1^ [44], indicating substantially higher overall PFAS burdens in some samples than those observed in the present study. A multinational survey of milk collected from 15 countries during 2020–2024 further showed that 26 of 32 investigated PFASs were detected in more than 40% of samples, although considerable differences in contamination profiles were observed among countries [45]. These studies indicate regional differences in PFAS occurrence in dairy products and provide broader context for the concentrations observed in the present sample set.

Potential sources of PFAS contamination in milk include contaminated feed, drinking water, environmental exposure, and food-processing materials. Studies conducted in China have shown that PFASs can occur simultaneously in feed, drinking water, and raw milk, and that PFOA may be efficiently transferred from environmental sources into milk [46,47]. These findings suggested that contamination of feed and drinking water, together with environmental exposure during dairy production, might represent important pathways for PFAS entry into milk. From a regulatory perspective, the detection of PFOA and other PFASs in commercial milk underscores the need for continued monitoring of dairy products. Although some jurisdictions have established regulatory limits for selected PFASs in certain food categories [48,49], the concentrations measured in this study cannot be directly interpreted as regulatory non-compliance, given that applicable limits vary across food categories and regulatory systems. The occurrence of PFOA and other PFASs in milk is also relevant to dietary exposure, particularly for children, for whom higher exposure risks have been reported in previous assessments. Based on the detected PFOA concentrations, the estimated daily intake (EDI) of PFOA from milk consumption was 0.123–0.873 and 0.925–6.55 ng·kg^−1^ body weight·day^−1^ for adults and children, respectively, under the assumed consumption scenarios (see Text S4) [44]. These estimates provide an indicative assessment of PFOA exposure from milk but do not represent total dietary exposure. Therefore, continued monitoring of PFASs in dairy products is warranted. The analysis of real samples thus demonstrates that the MSPE method established in this study is feasible for the determination of multiple long-chain PFAS contaminants in real milk samples.

## 4. Conclusions

In this study, an Fe_3_O_4_-PSA-based MSPE method coupled with LC-MS/MS was developed for the determination of 11 long-chain PFASs in milk. Key experimental parameters, including sample pH, adsorbent dosage, adsorption time, elution solvent, and elution time, were systematically optimized. The method showed satisfactory analytical performance, with LODs of 0.008–0.062 μg/kg, LOQs of 0.026–0.206 μg/kg, and acceptable recoveries and precision at three spiking levels. The combined zeta-potential, pH-dependent adsorption, chain-length-dependent adsorption, and XPS results probably support the involvement of electrostatic interactions, hydrophobic interactions, and hydrogen bonding in PFAS adsorption onto Fe_3_O_4_-PSA. Thermodynamic analysis further indicated that the adsorption process was thermodynamically favorable and exothermic within the investigated temperature range. The developed method enabled rapid magnetic separation with a relatively low amount of sorbent and without the use of conventional SPE cartridges. Application to commercial milk samples further demonstrated the feasibility of the method for trace long-chain PFAS determination in dairy matrices.

## Figures and Tables

**Figure 1:**
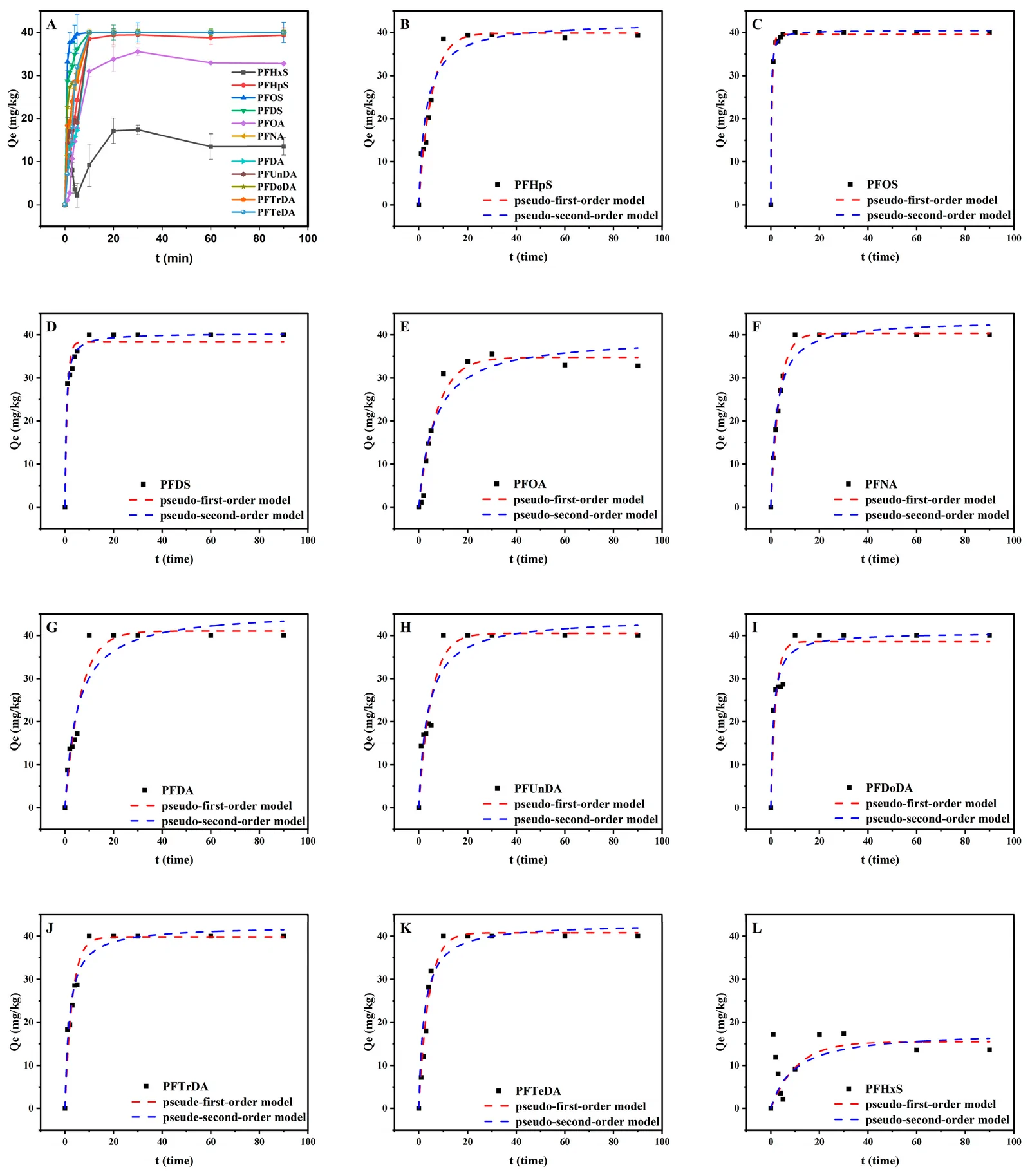
Adsorption kinetics of 11 PFASs on Fe_3_O_4_-PSA NPs and kinetic model fitting. (**A**) Adsorption kinetics of the 11 target PFASs. (**B**–**L**) Pseudo-first-order and pseudo-second-order kinetic model fitting for individual PFASs.

**Figure 2:**
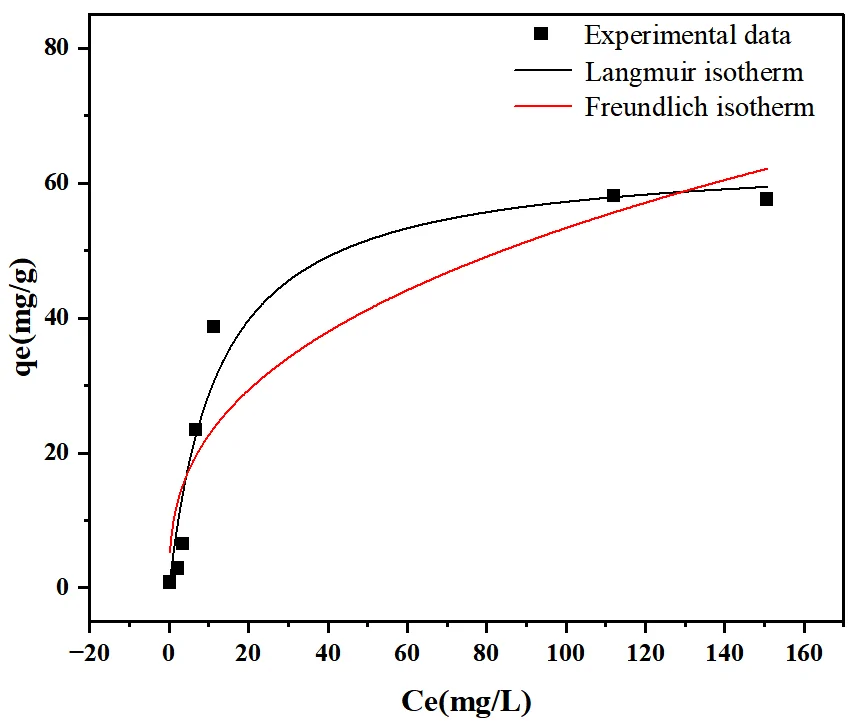
Langmuir and Freundlich adsorption isotherms of PFASs on Fe_3_O_4_-PSA.

**Figure 3:**
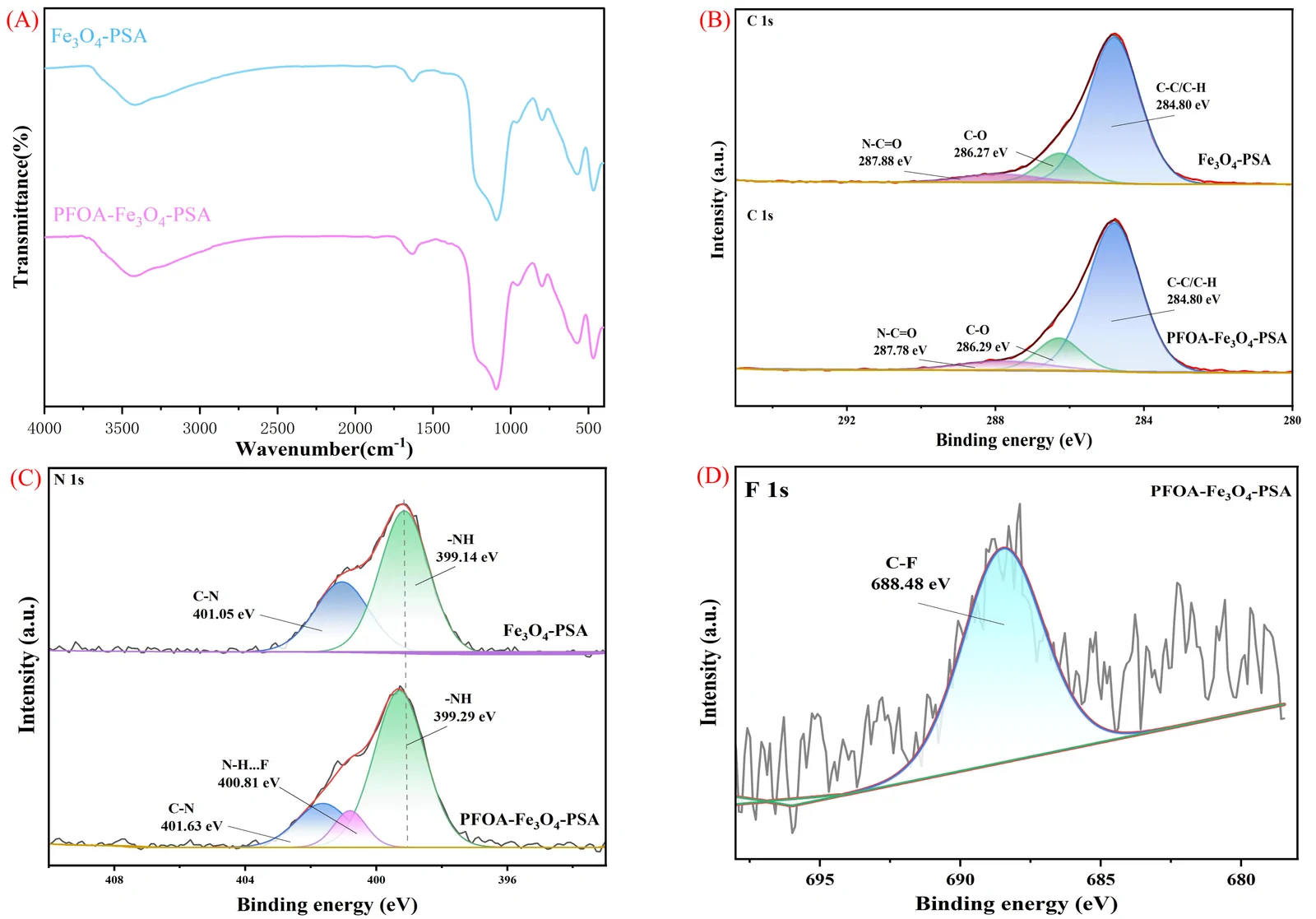
FTIR and XPS spectra of Fe_3_O_4_-PSA before and after PFOA adsorption. (**A**) FTIR spectra; (**B**) C 1s spectra; (**C**) N 1s spectra; and (**D**) F 1s spectrum.

**Figure 4:**
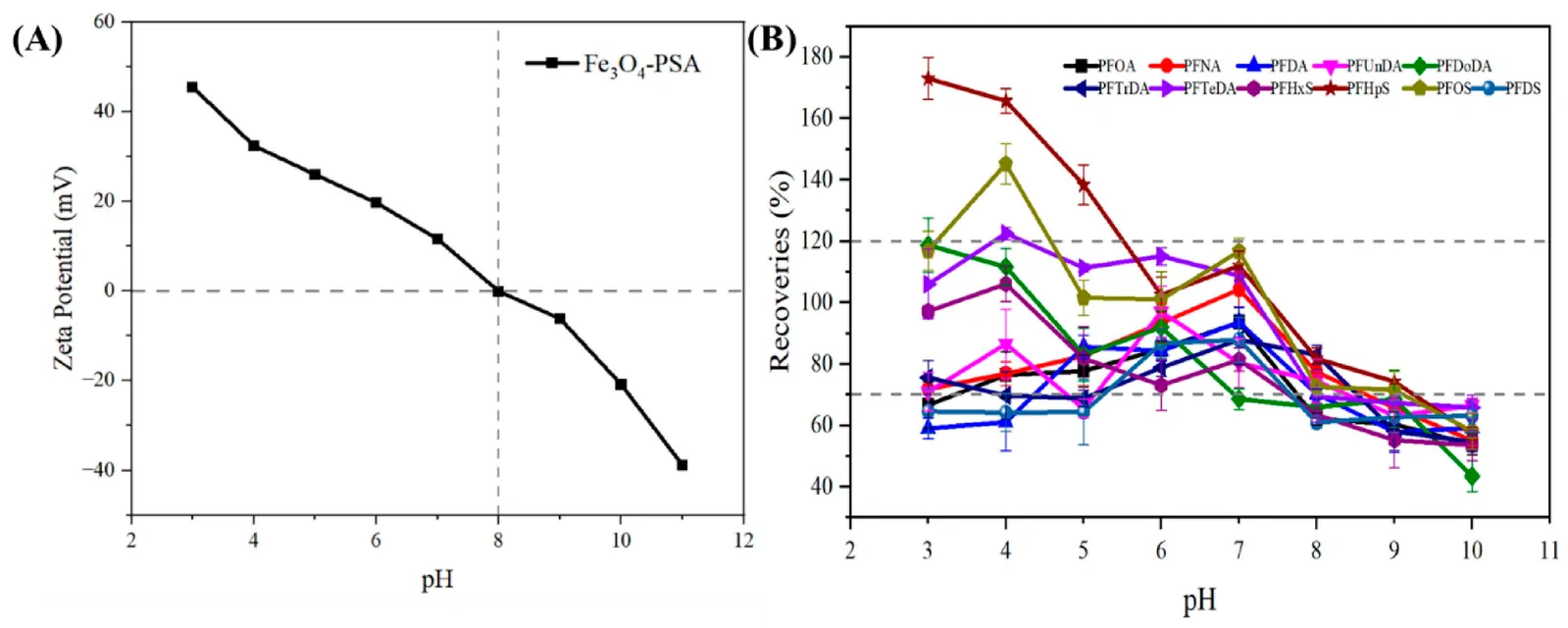
(**A**) Zeta potential of Fe_3_O_4_-PSA NPs under different pH values. (**B**) Influence of solution pH on the recoveries of PFASs.

**Figure 5:**
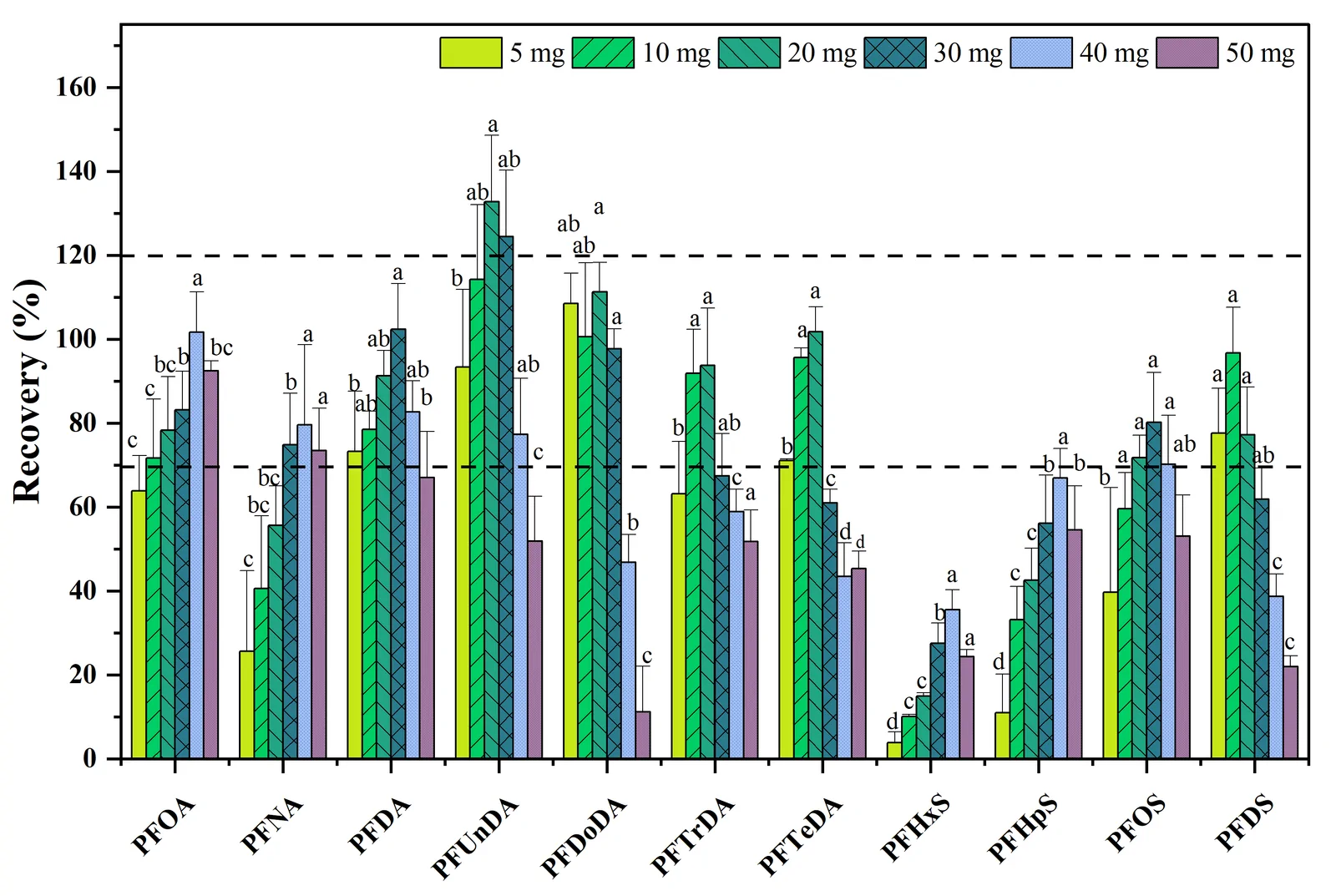
Influence of Fe_3_O_4_-PSA amount on the recoveries of PFASs. Different lowercase letters indicate significant differences among different adsorbent dosages for the same PFAS (*p *< 0.05).

**Figure 6:**
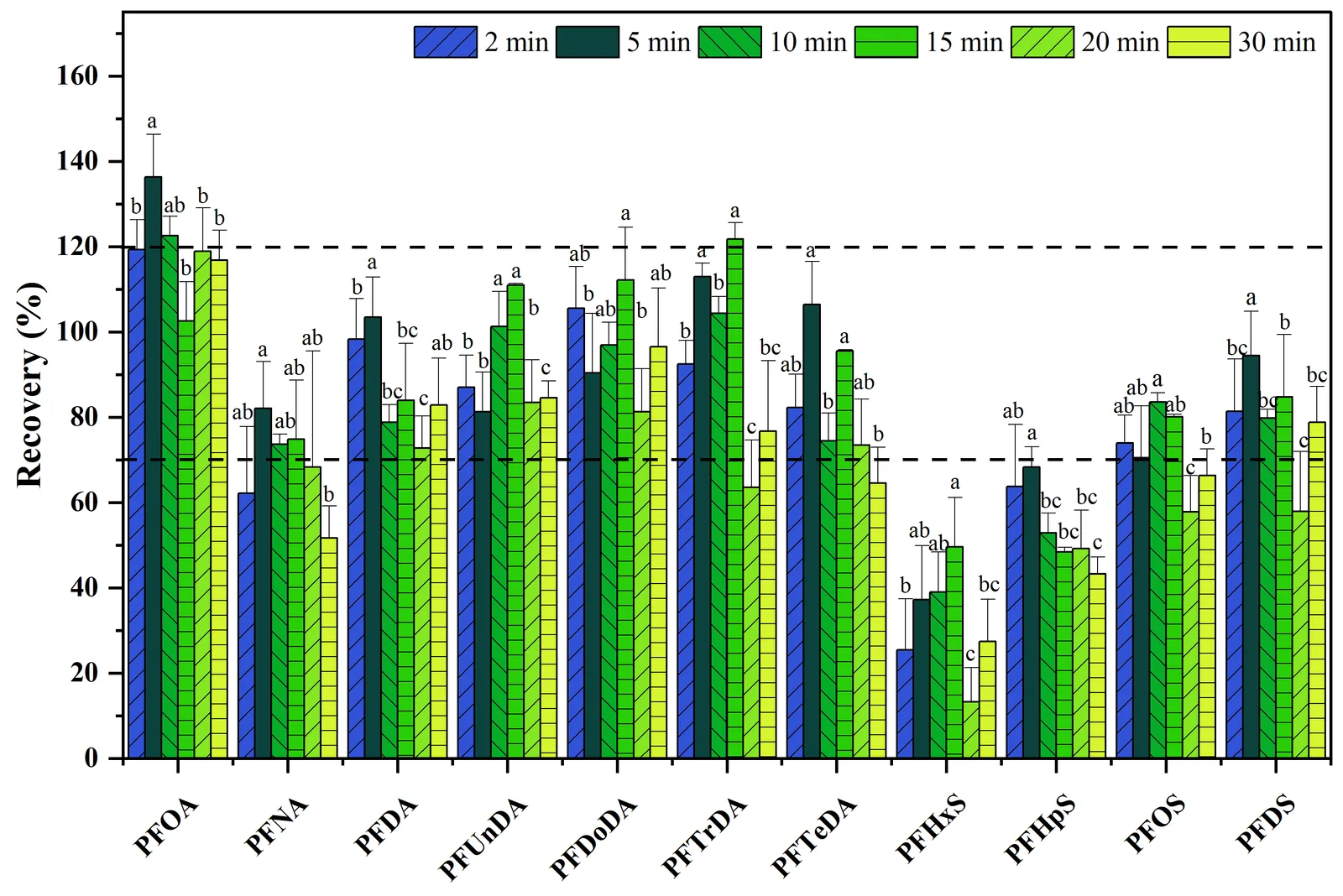
Influence of adsorption time on the recoveries of PFASs. Different lowercase letters indicate significant differences among different adsorption times for the same PFAS (*p *< 0.05).

**Figure 7:**
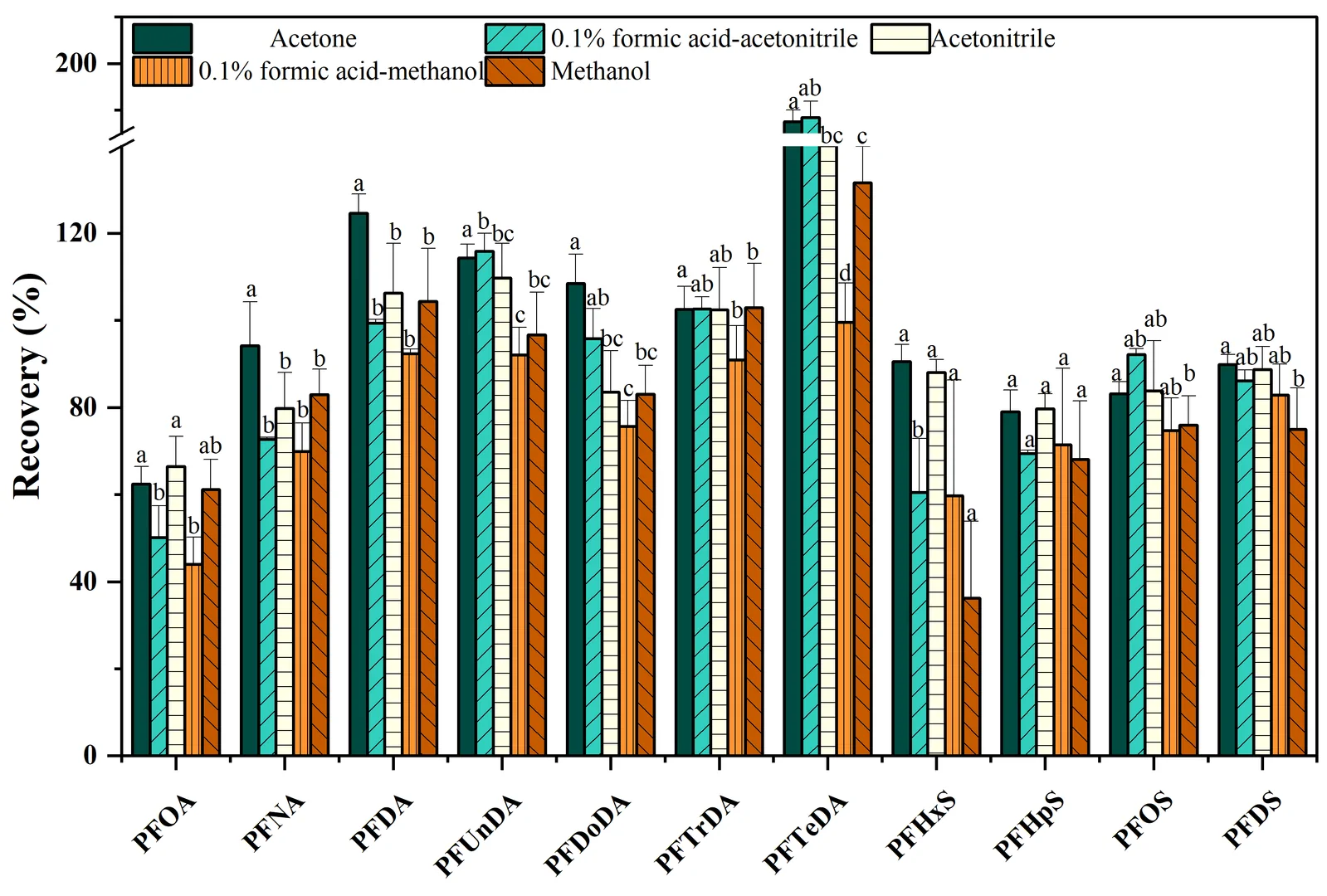
Influence of elution solvent type on the recoveries of PFASs. Different lowercase letters indicate significant differences among different eluents for the same PFAS (*p *< 0.05).

**Figure 8:**
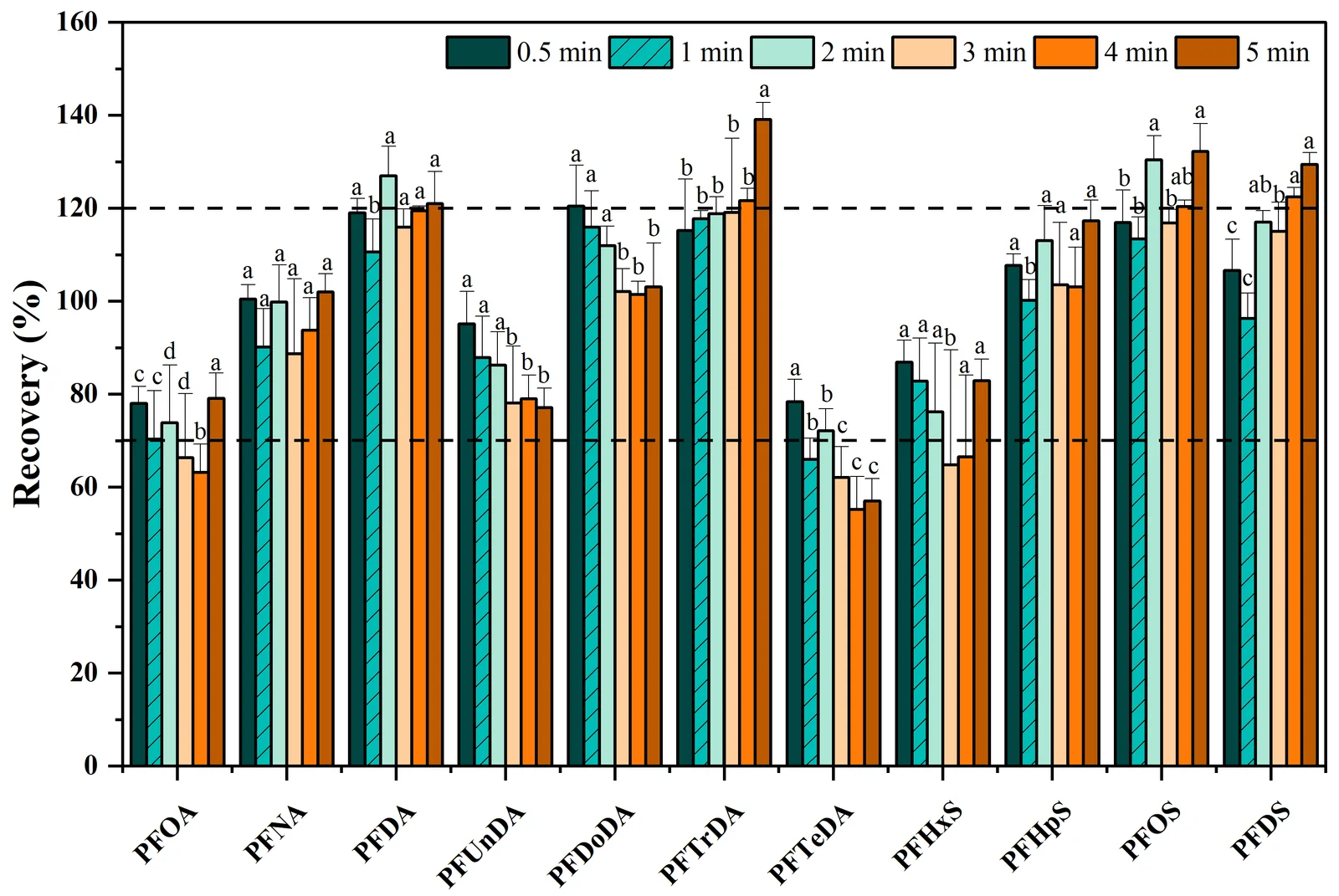
Influence of elution time on the recoveries of PFASs. Different lowercase letters indicate significant differences among different elution times for the same PFAS (*p *< 0.05).

**Figure 9:**
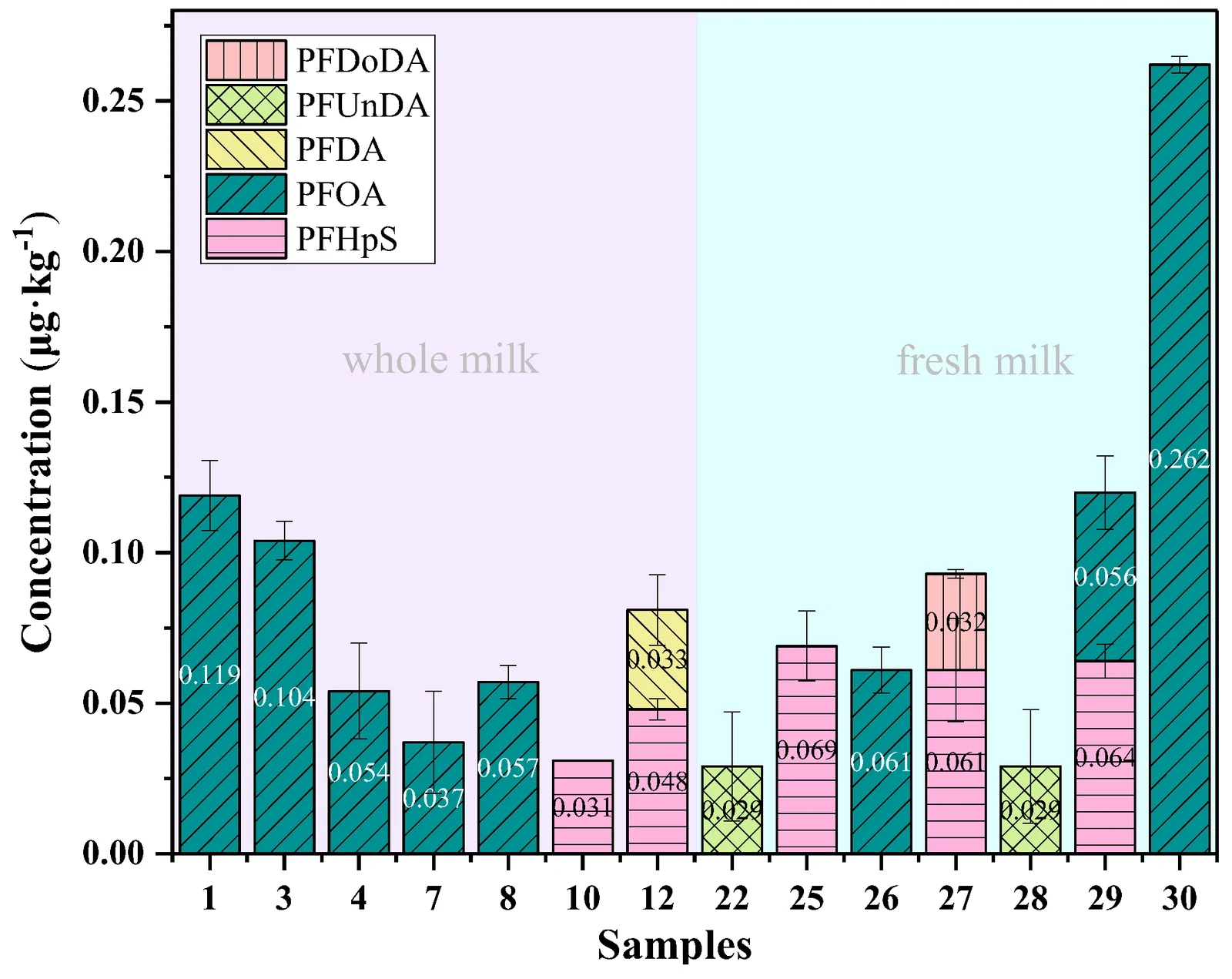
Concentration of PFASs in real milk samples.

**Table 1:** Mass spectrometry parameters for 11 PFAS test compounds and 2 ISs.

Number	PFASs	Internal Standards	Retention Time (min)	Precursor Ion (*m*/*z*)	Product Ion (*m*/*z*)	Collision Energy (eV)
1	PFOA	^13^C_2_-PFDA	8.694	413	369; 169	6; 16
2	PFNA	^13^C_2_-PFDA	9.164	463	419; 219	6; 14
3	PFDA	^13^C_2_-PFDA	9.558	513	469; 219	8; 14
4	PFUnDA	^13^C_2_-PFDA	9.901	563	519; 269	10; 18
5	PFDoDA	^13^C_2_-PFDA	10.198	613	569; 269	10; 18
6	PFTrDA	^13^C_2_-PFDA	10.461	663	619; 269	10; 24
7	PFTeDA	^13^C_2_-PFDA	10.695	713	669; 369	10; 18
8	PFHxS	^13^C_4_-PFOS	8.139	399	80; 99	46; 39
9	PFHpS	^13^C_4_-PFOS	8.679	449	80; 99	50; 40
10	PFOS	^13^C_4_-PFOS	9.129	499	99	52
11	PFDS	^13^C_4_-PFOS	9.856	599	80; 99	60; 58
12	^13^C_2_-PFDA	———	9.559	514.9	470; 219	10; 17
13	^13^C_4_-PFOS	———	9.130	502.9	80; 99	55; 42

Notes: In the column of product ions, the value on the left was quantitative ion, and the value on the right was qualitative ion.

**Table 2:** Method validation of the linearity, slope ratio and MLOQ of the PFASs in milk.

PFASs	Linear Equations	R^2^	^1^ Slope Ratio	LOD (μg·kg^−1^)	LOQ (μg·kg^−1^)
PFOA	^2^ y = 0.3669x + 0.0851	0.9986	0.96	0.029	0.096
	^3^ y = 0.3815x + 0.0867	0.9986			
PFNA	y = 0.3248x + 0.0532	0.9990	1.06	0.018	0.060
	y = 0.3073x + 0.0742	0.9983			
PFDA	y = 0.45x + 0.0982	0.9979	1.01	0.025	0.082
	y = 0.4464x + 0.0628	0.9981			
PFUnDA	y = 0.4683x + 0.0881	0.9991	1.03	0.008	0.026
	y = 0.4541x + 0.1568	0.9980			
PFDoDA	y = 0.4555x − 0.0245	0.9996	1.04	0.022	0.073
	y = 0.438x − 0.0062	0.9994			
PFTrDA	y = 0.4657x + 0.0506	0.9997	1.03	0.016	0.052
	y = 0.4501x − 0.0684	0.9972			
PFTeDA	y = 0.2396x + 0.0203	0.9996	0.98	0.020	0.066
	y = 0.2441x + 0.0656	0.9983			
PFHxS	y = 0.8786x − 0.0564	0.9988	1.06	0.014	0.045
	y = 0.8311x − 0.0592	0.9980			
PFHpS	y = 0.8293x + 0.0464	0.9992	1.01	0.028	0.093
	y = 0.82x − 0.0942	0.9981			
PFOS	y = 0.4755x − 0.0902	0.9989	1.09	0.062	0.206
	y = 0.4356x − 0.1287	0.9975			
PFDS	y = 0.6805x + 0.1027	0.9989	0.98	0.031	0.103
	y = 0.6843x + 0.027	0.9999			

Notes: ^1^ The value of slope ratio is calculated by the slope of the matrix-matched calibration curve to the slope of the standard calibration curve in solvent. ^2^ The first calibration curve for each PFAS represents the matrix-matched calibration curve of the milk sample. ^3^ The second calibration curve for each PFAS represents the calibration curve prepared in solvent.

**Table 3:** Comparison of reported methods for PFAS analysis.

Samples	Method	Sample Amount	Adsorbent		Extraction Time (min)	Solvent Consumption (mL)	LOD (ng·kg^−1^)	LOQ (ng·kg^−1^)	Recoveries (%)	Reference
			Type	Consumption (mg)						
Milk	QuEChERS	-	Fe_3_O_4_-SiO_2_ + ZrO_2_ + C18	140	5	-	4–79	10–260	71.7–116	[38]
Cow milk	QuEChERS	5 g	PSA + C18 + MgSO_4_	150 + 150 + 900	8	10	7.78–16.35	50–100	91.3–121.8	[39]
Food	MSPE	-	Fe_3_O_4_@CF-COF	-	-	-	3–19	-	73.5–118	[40]
Milk, tomato, fish	MSPE	-	Fe_3_O_4_@ZIF-8&F13	-	-	-	5–21	-	88.4–114.9	[41]
Milk	MSPE	2 g	Fe_3_O_4_-PSA	20	10	4	8–62	26–206	79–118	This work

## Data Availability

The original contributions presented in the study are included in the article/Supplementary Materials; further inquiries can be directed to the corresponding authors.

## Supplementary Materials

The following supporting information can be downloaded at https://www.mdpi.com/article/10.3390/foods15203622/s1, Text S1. Preparation of magnetic Fe_3_O_4_-PSA NPs; Text S2. Adsorption isotherm modelling; Text S3. Adsorption Thermodynamics; Text S4. Dietary exposure estimation; Table S1. Name, abbreviation, molecular formula, molecular weight and Pka for 11 PFAS test compounds and 2 ISs; Table S2. Product information of 30 real milk samples; Table S3. Kinetic parameters of the pseudo-first-order and pseudo-second-order adsorption models for 11 PFAS; Table S4. Thermodynamic parameters for PFAS adsorption onto Fe_3_O_4_-PSA at different temperatures; Table S5. Intra-day and inter-day recoveries and precision of PFAS in spiked milk sample at different concentration levels (n = 5); Figure S1. Representative MRM chromatograms of PFUnDA in (A) the matrix-matched standard, (B) the solvent standard, (C) the procedural blank, and (D) the matrix blank.

## References

[1] Jia S., Marques Dos Santos M., Li C., Snyder S.A. (2022). Recent advances in mass spectrometry analytical techniques for per- and polyfluoroalkyl substances (PFAS). Anal. Bioanal. Chem..

[2] Bolan N., Sarkar B., Yan Y., Li Q., Wijesekara H., Kannan K., Tsang D.C.W., Schauerte M., Bosch J., Noll H. (2020). Remediation of poly- and perfluoroalkyl substances (PFAS) contaminated soils—To mobilize or to immobilize or to destroy?. J. Hazard. Mater..

[3] Evich M.G., Davis M.J.B., McCord J.P., Acrey B., Awkerman J.A., Knappe D.R.U., Lindstrom A.B., Speth T.F., Tebes-Stevens C., Strynar M.J. (2022). Per- and polyfluoroalkyl substances in the environment. Science.

[4] Zhou Y., He Z., Tao Y., Xiao Y., Zhou T., Jing T., Zhou Y., Mei S. (2016). Preparation of a functional silica membrane coated on Fe_3_O_4_ nanoparticle for rapid and selective removal of perfluorinated compounds from surface water sample. Chem. Eng. J..

[5] OECD, FAO (2022). OECD-FAO Agricultural Outlook 2022–2031.

[6] Kowalczyk J., Ehlers S., Oberhausen A., Tischer M., Fuerst P., Schafft H., Lahrssen-Wiederholt M. (2013). Absorption, Distribution, and Milk Secretion of the Perfluoroalkyl Acids PFBS, PFHxS, PFOS, and PFOA by Dairy Cows Fed Naturally Contaminated Feed. J. Agric. Food Chem..

[7] Sznajder-Katarzyńska K., Surma M., Wiczkowski W., Cieślik E. (2019). The perfluoroalkyl substance (PFAS) contamination level in milk and milk products in Poland. Int. Dairy J..

[8] Wang J., Shi Y., Pan Y., Cai Y. (2010). Perfluorinated compounds in milk, milk powder and yoghurt purchased from markets in China. Chin. Sci. Bull..

[9] Young W.M., South P., Begley T.H., Diachenko G.W., Noonan G.O. (2012). Determination of Perfluorochemicals in Cow’s Milk Using Liquid Chromatography-Tandem Mass Spectrometry. J. Agric. Food Chem..

[10] Savoca D., Melfi R., Palumbo Piccionello A., Barreca S., Buscemi S., Arizza V., Arculeo M., Pace A. (2021). Presence and biodistribution of perfluorooctanoic acid (PFOA) in Paracentrotus lividus highlight its potential application for environmental biomonitoring. Sci. Rep..

[11] Barreca S., Busetto M., Colzani L., Clerici L., Marchesi V., Tremolada L., Daverio D., Dellavedova P. (2020). Hyphenated High Performance Liquid Chromatography–Tandem Mass Spectrometry Techniques for the Determination of Perfluorinated Alkylated Substances in Lombardia Region in Italy, Profile Levels and Assessment: One Year of Monitoring Activities During 2018. Separations.

[12] Domingo J.L., Nadal M. (2017). Per- and Polyfluoroalkyl Substances (PFASs) in food and human dietary intake: A review of the recent scientific literature. J. Agric. Food Chem..

[13] Yu Y., Xu D., Lu M., Zhou S., Peng T., Yue Z., Zhou Y. (2015). QuEChERs combined with online interference trapping LC-MS/MS method for the simultaneous determination of 20 polyfluoroalkane substances in dietary milk. J. Agric. Food Chem..

[14] Tittlemier S.A., Braekevelt E. (2011). Analysis of polyfluorinated compounds in foods. Anal. Bioanal. Chem..

[15] Keller J.M., Calafat A.M., Kato K., Ellefson M.E., Reagen W.K., Strynar M., O’Connell S., Butt C.M., Mabury S.A., Small J. (2010). Determination of perfluorinated alkyl acid concentrations in human serum and milk standard reference materials. Anal. Bioanal. Chem..

[16] Hill N.I., Becanova J., Lohmann R. (2022). A sensitive method for the detection of legacy and emerging per- and polyfluorinated alkyl substances (PFAS) in dairy milk. Anal. Bioanal. Chem..

[17] Iannone A., Carriera F., Passarella S., Fratianni A., Avino P. (2024). There’s Something in What We Eat: An Overview on the Extraction Techniques and Chromatographic Analysis for PFAS Identification in Agri-Food Products. Foods.

[18] Ogunbiyi O.D., Ajiboye T.O., Omotola E.O., Oladoye P.O., Olanrewaju C.A., Quinete N. (2023). Analytical approaches for screening of per- and poly fluoroalkyl substances in food items: A review of recent advances and improvements. Environ. Pollut..

[19] Taylor R.B., Sapozhnikova Y. (2022). Comparison and validation of the QuEChERSER mega-method for determination of per- and polyfluoroalkyl substances in foods by liquid chromatography with high-resolution and triple quadrupole mass spectrometry. Anal. Chim. Acta.

[20] Sun H., Yang Y., Shen H., Hao Q., Huang Q., Gao J., Liu X., Zhang H. (2024). Fluorine-functionalized magnetic amino microporous organic network for enrichment of perfluoroalkyl substances. J. Chromatogr. A.

[21] Mahpishanian S., Zhou M., Foudazi R. (2024). Magnetic amino-functionalized graphene oxide nanocomposite for PFAS removal from water. Environ. Sci. Adv..

[22] Ateia M., Alsbaiee A., Karanfil T., Dichtel W. (2019). Efficient PFAS Removal by Amine-Functionalized Sorbents: Critical Review of the Current Literature. Environ. Sci. Technol. Lett..

[23] Bano A., Prasad B., Dave H., Prasad K.S. (2026). Efficient adsorption of perfluorooctane sulfonate and perfluorobutane sulfonic acid from aqueous solution onto amine-functionalized diatomite granules. Sci. Rep..

[24] Qi P., Wang Z., Yang G., Shang C., Xu H., Wang X., Zhang H., Wang Q., Wang X. (2015). Removal of acidic interferences in multi-pesticides residue analysis of fruits using modified magnetic nanoparticles prior to determination via ultra-HPLC-MS/MS. Microchim. Acta.

[25] Zhang M., Li J., Zhang C., Wu Z., Yang Y., Li J., Fu F., Lin Z. (2020). In-situ synthesis of fluorinated magnetic covalent organic frameworks for fluorinated magnetic solid-phase extraction of ultratrace perfluorinated compounds from milk. J. Chromatogr. A.

[26] Abafe O.A., Macheka L.R., Olowoyo J.O. (2021). Confirmatory Analysis of Per and Polyfluoroalkyl Substances in Milk and Infant Formula Using UHPLC–MS/MS. Molecules.

[27] Liu Z., Zhao H., Wang J., Wang Z., Di S., Xu H., Wang Q., Wang X., Wang X., Qi P. (2022). Magnetic polymer particles as a highly efficient and facile cleanup adsorbent for multi-pesticide residues analysis in aquatic products. Ecotoxicol. Environ. Saf..

[28] Appleman T.D., Dickenson E.R., Bellona C., Higgins C.P. (2013). Nanofiltration and granular activated carbon treatment of perfluoroalkyl acids. J. Hazard. Mater..

[29] Yao Y., Zhu H., Li B., Hu H., Zhang T., Yamazaki E., Taniyasu S., Yamashita N., Sun H. (2014). Distribution and primary source analysis of per- and poly-fluoroalkyl substances with different chain lengths in surface and groundwater in two cities, North China. Ecotox. Environ. Saf..

[30] Zhou Y., Lian Y., Sun X., Fu L., Duan S., Shang C., Jia X., Wu Y., Wang M. (2019). Determination of 20 perfluoroalkyl substances in greenhouse vegetables with a modified one-step pretreatment approach coupled with ultra performance liquid chromatography tandem mass spectrometry(UPLC-MS-MS). Chemosphere.

[31] Qi P., Wang J., Liu Z., Wang Z., Xu H., Di S., Zhao H., Wang X. (2021). Integrated QuEChERS strategy for high-throughput multi-pesticide residues analysis of vegetables. J. Chromatogr. A.

[32] Bakhteeva I.A., Medvedeva I.V., Uimin M.A., Byzov I.V., Zhakov S.V., Yermakov A.E., Shchegoleva N.N. (2015). Magnetic sedimentation and aggregation of Fe_3_O_4_@SiO_2_ nanoparticles in water medium. Sep. Purif. Technol..

[33] Du Z., Deng S., Bei Y., Huang Q., Wang B., Huang J., Yu G. (2014). Adsorption behavior and mechanism of perfluorinated compounds on various adsorbents--a review. J. Hazard. Mater..

[34] Higgins P.C., Luthy G.R. (2006). Sorption of perfluorinated surfactants on sediments. Environ. Sci. Technol..

[35] Rayne S., Forest K. (2009). Perfluoroalkyl sulfonic and carboxylic acids: A critical review of physicochemical properties, levels and patterns in waters and wastewaters, and treatment methods. J. Environ. Sci. Health Part A.

[36] European Commission (2010). Commission Recommendation of 17 March 2010 on the monitoring of perfluoroalkylated substances in food (2010/161/EU). Off. J. Eur. Union L.

[37] Capriotti A.L., Cavaliere C., Cavazzini A., Foglia P., Lagana A., Piovesana S., Samperi R. (2013). High performance liquid chromatography tandem mass spectrometry determination of perfluorinated acids in cow milk. J. Chromatogr. A.

[38] Shi R., Liu L., Liu X., Liu Z., Liu J., Wang J., Di S., Qi P., Wang X. (2023). Integrated QuEChERS combined with LC–MS/MS for high-throughput analysis of per- and polyfluoroalkyl substances in milk. Anal. Bioanal. Chem..

[39] Gallocchio F., Moressa A., Zonta G., Angeletti R., Lega F. (2022). Fast and Sensitive Analysis of Short- and Long-Chain Perfluoroalkyl Substances in Foods of Animal Origin. Molecules.

[40] Gong J., Zhang X., Liang R., Ma J., Yang N., Cai K., Wu J., Xie Z., Zhang S., Chen Y. (2024). Rapidly enrichment and detection of per-and polyfluoroalkyl substances in foods using a novel bifunctional covalent organic framework. Food Chem..

[41] Liu T., Sang C., Liu Y., Shao B., He G. (2025). Efficient extraction of perfluoroalkyl carboxylic acids from food samples using a fluorinated magnetic metal-organic framework adsorbent. Food Chem..

[42] Xiao K., Li X., Xu N., Wang X., Hao L., Bao H., Zhang L., Shi Y., Cai Y. (2024). Carry-over rate of per- and polyfluoroalkyl substances to raw milk and human exposure risks in different regions of China. Sci. Total Environ..

[43] Curci D., Sundaram T.S., Ghidini S., Arioli F. (2025). What We Know About per- and Polyfluoroalkyl Contamination Levels in Milk. A Review from the Last Decade. Foods.

[44] Macheka L.R., Olowoyo J.O., Mugivhisa L.L., Abafe O.A. (2020). Determination and assessment of human dietary intake of per and polyfluoroalkyl substances in retail dairy milk and infant formula from South Africa. Sci. Total Environ..

[45] Li X., Liu Y., Li T., Zhao N., Pei X., Wang R., Wu M., Han X., Liu X., Wang P. (2025). Unraveling Legacy and Emerging PFAS Characteristics in Milk across 15 Countries (2020–2024): Features, Temporal Trend,s and Risk Priority. Environ. Sci. Technol..

[46] Liu Y., Zhang Q., Li Y., Hao Y., Li J., Zhang L., Wang P., Yin Y., Zhang S., Li T. (2022). Occurrence of per- and polyfluoroalkyl substances (PFASs) in raw milk and feed from nine Chinese provinces and human exposure risk assessment. Chemosphere.

[47] Hossini H., Massahi T., Parnoon K., Nouri M. (2025). Per-and polyfluoroalkyl substances (PFAS) in milk and dairy products: A literature review of the occurrence, contamination sources, and health risks. Food Addit. Contam. Part A.

[48] Schrenk D., Bignami M., Bodin L., Chipman J.K., del Mazo J., Grasl-Kraupp B., Hogstrand C., Hoogenboom L., Leblanc J.C., EFSA Panel on Contaminants in the Food Chain (EFSA CONTAM Panel) (2020). Risk to human health related to the presence of perfluoroalkyl substances in food. EFSA J..

[49] European Commission (2023). Commission Regulation (EU) 2023/915 of 25 April 2023 on Maximum Levels for Certain Contaminants in Food and Repealing REGULATION (EC) No 1881/2006.

