# Supplementary material for: Sensitive and Rapid Determination of Long-Chain Per- and Polyfluoroalkyl Substances in Milk Using Fe3O4-PSA-Based Magnetic Solid-Phase Extraction Coupled with LC-MS/MS
Source: Foods. 2026 Oct 12;15(20):3622. doi: 10.3390/foods15203622 (PMC13703254; doi:10.3390/foods15203622)
Supplement: Supplementary file 1 [file foods-15-03622-s001.zip › foods-4488459-supplementary.pdf]

Supplementary material

Sensitive and Rapid Determination of Long-Chain Per and Polyfluoroalkyl Substances in Milk Using Fe_3O_4-PSA-Based Magnetic Solid-Phase Extraction Coupled with LC-MS/MS

Tiantian Niu [a,b], Nannan Wu [b], Ranran Shi [b], Zhenzhen Liu [b], Siying Che [a,*], Xinquan Wang [b,*], Peipei Qi [b,*]

Author Affiliation(s)

[a] College of Chemical Engineering, Zhejiang University of Technology, Hangzhou 310014, China

State Key Laboratory for Quality and Safety of Agro-Products & Institute of Agro-product Safety and Nutrition, Zhejiang Academy of Agricultural Sciences, Hangzhou 310021, PR China.

[*] Contact information for Corresponding Author

Corresponding Author: Siying Che, Xinquan Wang, Peipei Qi

Address:

Siying Che, Zhejiang University of Technology, Hangzhou 310014, China, chesiying@zjut.edu.cn

Xinquan Wang, Zhejiang Academy of Agricultural Sciences, Hangzhou 310021, PR China. Email: wangxinquan212@163.com.

Peipei Qi, Zhejiang Academy of Agricultural Sciences, Hangzhou 310021, PR China. E-mail: qipeipei@zaas.ac.cn.

Text S1 Preparation of magnetic Fe_3O_4-PSA NPs

Fe_3O_4-PSA NPs was synthesized according to a published method in our lab (Qi et al., 2015). Firstly, magnetic Fe_3O_4 NPs were synthesized by solvent thermal process. 0.4 g sodium citrate was dissolved in 72 mL glycol and placed in a beaker. 16 mL solution A (5.0 g $FeCl_3 \cdot 6H_2O$ was dissolved in 100 ml glycol) and solution B (10 g sodium acetate dissolved in 72 ml glycol) were added successively. After being thoroughly stirred for 30 min, the mixture was sealed in a Teflon-lined stainless-steel autoclave. Keep at 210°C for 10 h and cool to room temperature. The resulting product was collected with external magnetic field (a rubidium iron boron strong magnet) and washed several times with water and ethanol. The final product was dried under vacuum at 60°C overnight.

Fe_3O_4-SiO_2 NPs were prepared by modifying magnetic Fe_3O_4 NPs with tetraethyl orthosilicate (TEOS). 1g Fe_3O_4 NPs was dissolved in 100 mL of water and added to a flask containing 2 g hexadecyltrimethylammonium bromide and 200mL of water. Add 50 mg of aqueous ammonia and stirred thoroughly. 2.5 mL TEOS and 1.4 mL ethanol were added drop by drop under a mild nitrogen stream and then reacted at 60°C for 12 h. After reaction, the product was separated by external magnetic field and was washed by acetone and ethanol.

0.6 g Fe_3O_4-SiO_2 and 250 mL absolute toluene were added to a 500 mL flask and sonicated for 10 min under a nitrogen stream. 3 mL PSA-TMOS in absolute toluene and 1 mL dimethylamine were added and sonicated for 5 min, followed by stirring for 30 min at room temperature. The reaction was kept for 6 h at 65 °C. The obtained

products were separated by external magnetic field and washed with acetone, ethanol and water. After vacuum drying overnight, Fe_3O_4-PSA NPs were obtained.

Text S2 Adsorption isotherm modelling

The adsorption isotherm experiments were conducted to investigate the adsorption behavior of PFAS on Fe_3O_4-PSA at equilibrium. Briefly, a fixed amount of Fe_3O_4-PSA was added to PFAS solutions with different initial concentrations under the optimized adsorption conditions. After an adsorption time sufficient to reach equilibrium, the adsorbent was magnetically separated, and the residual PFAS concentrations in the supernatants were determined by LC-MS/MS. The equilibrium adsorption capacity (q_e) was calculated according to Eq. (S1):

$$q_e = \frac{(C_0 - C_e)V}{m} \quad \text{(S1)}$$

where q_e is the equilibrium adsorption capacity(μg/g),C_0 and C_e are the initial and equilibrium concentrations of PFAS in the solution (μg/L), respectively, V is the solution volume (L), and m is the mass of Fe_3O_4-PSA (g).

The equilibrium adsorption data were further fitted using the nonlinear Langmuir and Freundlich isotherm models. The Langmuir model was expressed as:

$$qe = \frac{q_m K_L C_e}{1 + K_L C_e} \quad \text{(S2)}$$

where qm (mg/g) is the maximum adsorption capacity and KL (L/mg) is the Langmuir adsorption constant.

The Freundlich model was expressed as:

$$q_e = K_F C_e^{1/n} \quad \text{(S3)}$$

where K_F is the Freundlich adsorption constant and nn is the Freundlich heterogeneity parameter. The goodness of fit of the two models was evaluated using the coefficient of determination (R^2).

Text S3 Adsorption Thermodynamics

The adsorption thermodynamics of PFAS onto Fe_3O_4-PSA were investigated at three different temperatures. A fixed amount of Fe_3O_4-PSA was added to PFAS solutions with a fixed initial concentration, and the suspensions were shaken in a temperature-controlled shaker at different temperatures (288.15, 298.15 and 308.15 K). After reaching adsorption equilibrium, the Fe_3O_4-PSA was magnetically separated, and the equilibrium concentration (C_e) of PFAS in the supernatant was determined by LC-MS/MS. The equilibrium adsorption capacity q_e (μg/g) was obtained using Eq.(S1).

The thermodynamic parameters, such as Gibbs free energy ΔG_0 (kJ mol^{-1}), standard enthalpy change ΔH_0 (kJ mol^{-1}) and standard entropy change ΔS_0 (kJ $mol^{-1}K^{-1}$), were calculated according to Eq. (S2-S3):

$$\Delta G^0 = -RT\ln K_0 \tag{S2}$$

$$\ln K_0 = -\frac{\Delta H^0}{RT} + \frac{\Delta S^0}{R} \tag{S3}$$

K_0 was the ratio of equilibrium adsorption capacity (q_e) to the equilibrium concentration (C_e). R was universal gas constant (8.314x10^{-3} kJ $mol^{-1}K^{-1}$). T (K) is the absolute temperature. The plot of $\ln K^0$ versus 1/T was constructed according to the Van’t Hoff equation. The values of ΔH^0 and ΔS^0were calculated from the slope and intercept of the linear fitting curve.

Text S4 Dietary exposure estimation

To contextualize the health significance of the detected PFOA levels, a preliminary dietary exposure assessment was performed. Daily PFOA intake was estimated using the equation:

$$\mathrm{EDI} = \frac{\mathrm{C} \times \mathrm{CR}}{\mathrm{BW}}$$

where EDI is the estimated daily intake ($\mathrm{ng \cdot kg^{-1}\ bw \cdot day^{-1}}$), C is the PFOA concentration in milk ($\mathrm{ng \cdot kg^{-1}}$), CR is the daily milk consumption rate ($\mathrm{kg \cdot day^{-1}}$), and BW is body weight (kg).

The PFOA concentration range detected in this study was 0.037–0.262 $\mathrm{ng \cdot g^{-1}}$. For the preliminary exposure estimation, a daily milk consumption of 0.2 $\mathrm{kg \cdot day^{-1}}$ and a body weight of 60 kg were assumed for adults, while a daily milk consumption of 0.5 $\mathrm{kg \cdot day^{-1}}$ and a body weight of 20 kg were assumed for children.

The calculated EDI values represent PFOA exposure from milk consumption only and do not account for exposure from other dietary sources, drinking water, or other environmental pathways. Therefore, these values should be considered indicative estimates rather than comprehensive dietary exposure or health risk assessments.

Table S1. Name, abbreviation, molecular formula, molecular weight and Pka for 11 PFAS test compounds and 2 ISs

NO.	Analytes	Abbreviation	Molecular Formula	Molecular Weight	Pka
1	Perfluorooctanoic acid	PFOA	$C_8HF_{15}O_2$	414.07	3.8
2	Perfluorononanoic acid	PFNA	$C_9HF_{17}O_2$	464.08	0.21
3	Perfluorodecanoic acid	PFDA	$C_{10}HF_{19}O_2$	514.08	-5.2
4	Perfluoroundecanoic acid	PFUnDA	$C_{11}HF_{21}O_2$	564.00	-5.2
5	Perfluorododecanoic acid	PFDoDA	$C_{12}HF_{23}O_2$	614.10	-5.2
6	Perfluorotridecanoic acid	PFTrDA	$C_{13}HF_{25}O_2$	664.11	-5.2
7	Perfluorotetradecanoic acid	PFTeDA	$C_{14}HF_{27}O_2$	714.11	-5.2
8	Perfluorohexanesulfonic acid	PFHxS	$C_6HF_{13}O_3S$	400.11	0.14
9	Perfluoroheptanesulfonic acid	PFHpS	$C_7HF_{15}O_3S$	450.12	-
10	Perfluorooctane sulfonic acid	PFOS	$C_8HF_{17}O_3S$	500.13	<1.0
11	Perfluorodecane Sulfonic acid	PFDS	$C_{10}HF_{21}O_3S$	600.14	-3.24
12	Perfluoro-*n*-[1,2-$^{13}C_2$] decanoic acid	$^{13}C_2$-PFDA	$^{13}C_2{}^{12}C_8HF_{19}O_2$	516.07	-
13	Sodium perfluoro-1-[1,2,3,4-$^{13}C_4$] octanesulfonate	$^{13}C_4$-PFOS	$^{13}C_4{}^{12}C_4F_{17}SO_3Na$	526.08	-

Table S2. Product information of 30 real milk samples

NO.	Type of production	Ingredients	Protein content /100g	Fat content/100g
1	Full fat sterilized milk	raw milk	3.2 g	4.0 g
2	Full fat sterilized milk	raw milk	3.2 g	3.8 g
3	Ultra-high temperature sterilized milk	raw milk	3.2 g	3.8 g
4	Full fat sterilized milk	raw milk	3.0 g	3.6 g
5	Full fat sterilized milk	raw milk	3.8 g	4.6 g
6	Full fat sterilized milk	raw milk	3.8 g	4.6 g
7	Full fat sterilized milk	raw milk	4.0 g	4.7 g
8	Ultra-high temperature sterilized milk	raw milk	3.3 g	3.6 g
9	Full fat sterilized organic milk	Organic raw milk	3.8 g	4.6 g
10	Full fat sterilized milk	Organic raw milk	3.8 g	4.6 g
11	Full fat sterilized milk	raw milk	3.6 g	4.4 g
12	Full fat sterilized milk	raw milk	3.6 g	4.4 g
13	Full fat sterilized milk	raw milk	3.0 g	3.8 g
14	Full fat sterilized milk	raw milk	3.0 g	3.6 g
15	Pasteurized milk	raw milk	3.1 g	3.6 g
16	Full fat sterilized milk	raw milk	3.0 g	3.3 g
17	Partially degreased sterilized milk	raw milk	3.6 g	1.3 g
18	Defatted sterilized pure milk	raw milk	3.2 g	0.00
19	Ultra-high temperature sterilized milk	raw milk	3.4 g	0.00
20	Fat-free sterilized milk	raw milk	3.6 g	0.00
21	Fat-free sterilized organic pure milk	Organic raw milk	3.8 g	0.00
22	Pasteurized milk	raw milk	3.4 g	3.4 g
23	Pasteurized milk	raw milk	3.4 g	3.9 g
24	Pasteurized milk	raw milk	3.1 g	3.5 g
25	Pasteurized milk	raw milk	3.6 g	3.9 g
26	Pasteurized milk	raw milk	3.8 g	4.6 g
27	Pasteurized milk	raw milk	3.6 g	3.9 g
28	Pasteurized milk	raw milk	3.8 g	4.6 g
29	Full fat pasteurized milk	Organic raw milk	3.5 g	4.2 g
30	Full fat pasteurized milk	raw milk	3.6 g	4.2 g

Table S3. Kinetic parameters of the pseudo-first-order and pseudo-second-order adsorption models for 11 PFAS.

analyte	Kinetic models					
	Pseudo-first-order			Pseudo-second-order		
	Q_{e1}(mg/g)	k_1 (min^{-1})	R^2	Q_{e2}(mg/g)	k_2(g/mg/min)	R^2
PFHpS	39.849	0.197	0.973	42.445	0.008	0.955
PFOS	39.536	1.775	0.997	40.512	0.126	0.998
PFDS	38.348	1.014	0.950	40.352	0.047	0.987
PFOA	34.770	0.140	0.961	39.595	0.004	0.912
PFNA	40.297	0.289	0.996	43.429	0.009	0.975
PFDA	41.007	0.159	0.954	45.852	0.004	0.917
PFUnDA	40.490	0.198	0.932	44.158	0.006	0.912
PFDoDA	38.587	0.500	0.900	40.694	0.022	0.951
PFTrDA	39.820	0.334	0.960	42.327	0.012	0.966
PFTeDA	40.770	0.246	0.977	42.940	0.010	0.949
PFHxS	15.544	0.094	0.860	18.038	0.006	0.757

Table S4. Thermodynamic parameters for PFAS adsorption onto Fe_3O_4-PSA at different temperatures.

Adsorbent	Temperature(K)	**Thermodynamics**		
		ΔG(kJ/mol)	ΔH(kJ/mol)	ΔS(J/mol/K)
	288	-3.125		
Fe_3O_4-PSA	298	-1.693	-48.88	-158.56
	308	-0.01419		

Table S5. Intra-day and inter-day recoveries and precision of PFAS in spiked milk sample at different concentration levels (n = 5)

PFCs	Intra-day recovery (RSD, %)			Inter-day recovery (RSD, %)		
	0.5 μg/kg	5 μg/kg	20 μg/kg	0.5 μg/kg	5 μg/kg	20 μg/kg
PFOA	80.5 (17)	94.1 (16)	110 (8.4)	79.5 (12)	98.4 (6.8)	94.6 (4.8)
PFNA	111 (12)	118 (2.7)	91.6 (6.9)	80.7 (19)	92.3 (9.1)	107 (9.7)
PFDA	116 (7.6)	101 (7.0)	108 (7.5)	107 (11)	99.7 (6.9)	114 (8.2)
PFUnDA	118 (11)	97.8 (6.9)	102 (3.7)	107 (8.4)	107 (8.8)	120 (10)
PFDoDA	112 (14)	98.0 (8.2)	117 (6.6)	98.5 (9.3)	110 (11)	110 (9.1)
PFTrDA	99.0 (9.4)	98.7 (8.2)	104 (9.6)	90.0 (9.3)	114 (3.5)	99.6 (16)
PFTeDA	102 (6.3)	90.2 (4.8)	113 (6.9)	120 (8.3)	107 (9.0)	113 (7.9)
PFHxS	86.1 (9.5)	79.4 (8.4)	104 (7.3)	106 (4.5)	86.0 (12)	87.3 (9.7)
PFHpS	93.7 (11)	112 (3.8)	95.7 (7.7)	120 (11)	98.3 (1.6)	96.5 (11)
PFOS	107 (8.0)	117 (14)	107 (7.3)	76.3 (16)	99.7 (7.6)	104 (4.5)
PFDS	102 (11)	110 (4.1)	116 (3.5)	114 (5.6)	109 (5.6)	108 (7.9)

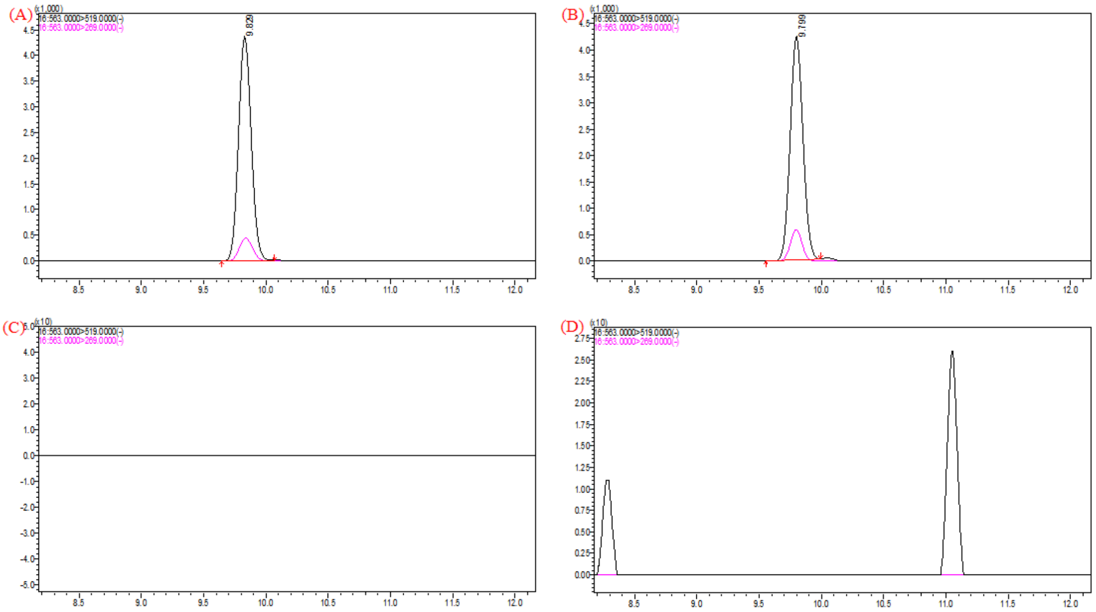

Figure S1. Representative MRM chromatograms of PFUnDA in (A) the matrix-matched standard, (B) the solvent standard, (C) the procedural blank, and (D) the matrix blank.
